# Infection of Human Tracheal Epithelial Cells by H5 Avian Influenza Virus Is Regulated by the Acid Stability of Hemagglutinin and the pH of Target Cell Endosomes

**DOI:** 10.3390/v12010082

**Published:** 2020-01-09

**Authors:** Tomo Daidoji, Junichi Kajikawa, Yasuha Arai, Yohei Watanabe, Ryohei Hirose, Takaaki Nakaya

**Affiliations:** 1 Department of Infectious Diseases, Graduate School of Medical Science, Kyoto Prefectural University of Medicine, Kyoto 602-8566, Japan; 2 Department of Molecular Gastroenterology and Hepatology, Graduate School of Medical Science, Kyoto Prefectural University of Medicine, Kyoto 602-8566, Japan

**Keywords:** avian influenza virus, tracheal epithelial cells, hemagglutinin, endosomal pH

## Abstract

Despite the possible relationships between tracheal infection and concomitant infection of the terminal part of the lower respiratory tract (bronchioles/alveoli), the behavior of avian influenza viruses (AIVs), such as H5N1, in the conducting airways is unclear. To examine the tropism of AIVs for cells lining the conducting airways of humans, we established human tracheal epithelial cell clones (HTEpC-Ts) and examined their susceptibility to infection by AIVs. The HTEpC-Ts showed differing susceptibility to H5N1 and non-zoonotic AIVs. Viral receptors expressed by HTEpC-Ts bound all viruses; however, the endosomal pH was associated with the overall susceptibility to infection by AIVs. Moreover, H5N1 hemagglutinin broadened viral tropism to include HTEpC-Ts, because it had a higher pH threshold for viral–cell membrane fusion. Thus, H5N1 viruses infect human tracheal epithelial cells as a result of their higher pH threshold for membrane fusion which may be one mechanism underlying H5N1 pathogenesis in human airway epithelia. Efficient replication of H5N1 in the conducting airways of humans may facilitate infection of the lower respiratory tract.

## 1. Introduction

Since the first case of human infection in Hong Kong in 1997, H5N1 viruses have remained a serious threat to public health worldwide, even though recent reports show that other subtypes of avian influenza viruses (AIVs), such as H5N6, H6N1, H7N2, H7N3, H7N4, H7N7, H7N9, H9N2, H10N7, and H10N8, can also infect humans [1,2,3,4,5,6,7,8,9,10,11,12,13,14,15,16]. AIVs are believed to typically infect human bronchiolar and alveolar epithelial cells [17,18]; however, viral RNA and/or viral antigens have been detected in the tracheal tissue or tracheal aspirates of H5N1-infected patients as well as in alveolar pneumocytes [19,20,21,22,23,24]. In addition, infectious viruses have been isolated from the tracheal aspirates of patients infected with H5N1 [21,25].

Collectively, these studies suggest that cells lining the human conducting airways (e.g., trachea) are susceptible to infection by H5N1. They also suggest a possible relationship between tracheal infection and concomitant infection of the terminal part of the lower respiratory tract (LRT) (i.e., bronchioles/alveoli); this is because the replication of respiratory viruses (e.g., influenza viruses) in the conducting airways can lead to infection of the lower respiratory region which can result in acute respiratory distress syndrome (ARDS). Therefore, to fully understand the pathogenesis of AIVs in humans, we need to know the extent to which AIVs infect the conducting airways of the human respiratory tract. However, the behavior of AIVs, such as H5N1, in the conducting airways is unclear.

Usually, cell lines, such as Madin–Darby canine kidney (MDCK) cells and A549 cells, both of which are susceptible to influenza viruses, are used to examine viral tropism and related pathogenesis [26,27,28,29]. These model cells are very useful for examining the transportation of the viral genome within host cells or for investigating the interaction between cellular (host) factors and the virus. However, these cell lines are less suitable for investigating the mechanisms underlying AIV infection of human respiratory epithelial cells, especially those in the conducting airways, because either they did not originate in humans or are not found in the conducting airways (e.g., MDCK cells are derived from dog; A549 cells are a cancer cell line derived from human type II pneumocytes). Therefore, to examine the mechanism by which AIVs infect the conducting airways in humans, we need to use an appropriate model cell. Because airway epithelial primary cells, such as NHBEs (normal human bronchial epithelial cells) [30,31] or SAECs (small airway epithelial cells) [32,33], are potentially good models for investigating this phenomenon in the human body, these cells have been used for pathogenesis studies of influenza viruses [30,31,32,33]. However, the weakness of primary cells is that the number of subcultures is very limited. To overcome this constraint, we previously established a human respiratory cell line named SAEC-T [34] which is derived from primary bronchioles; we then analyzed the susceptibility of these cells to infection by AIVs and the mechanisms that determine viral infectivity of the terminal part of the human LRT. However, the AIV susceptibility of cells in other regions of the airway tract has not been studied; therefore, the mechanisms underlying viral infection of the LRT have not been revealed completely.

In this study, we transformed primary human tracheal epithelial cells (HTEpCs) with SV40 large T-antigen and isolated several clonal cell lines (named HTEpC-Ts). We then used these lines to evaluate the tropism of AIVs for human conducting airways and examined the resulting pathogenic effects. We focused on the susceptibility of cells to infection by currently circulating H5N1 (zoonotic) and previously circulating (non-zoonotic) AIVs. In addition, we identified the mechanism responsible for the susceptibility of HTEpC-Ts to infection by current H5N1 and non-zoonotic AIVs.

## 2. Materials and Methods

### 2.1. Ethics Statement

Primary human cells (HTEpCs) were purchased from PromoCell Corp., Heidelberg, Germany (see Section 2.2). All donors (or an authorized or legal agent) completed and signed an informed consent form. All experiments described herein were performed in vitro using these primary human cells and other cell lines. All experiments using recombinant DNA were conducted under the relevant Japanese laws and were approved by the Biological Safety Committee of Kyoto Prefectural University of Medicine (Approval number 29-120; 30-146) after risk assessments were conducted by the Living Modified Organisms Committee of Kyoto Prefectural University of Medicine and (when required) by the Ministry of Education, Culture, Sports, Science, and Technology of Japan.

### 2.2. Viruses and Cells

The A/Beijing/262/95 (H1N1) (Beijing (H1N1)), A/Panama/2007/99 (H3N2) (Panama (H3N2)), A/crow/Kyoto/53/04 (H5N1) (Cw/Ky (H5N1)), A/chicken/Egypt/CL6/07 (H5N1) (Ck/Eg (H5N1)), A/duck/Hong Kong/820 (H5N3) (Dk/Hk (H5N3)), and A/turkey/Ontario/7732/66 (H5N9) (Tk/Ont (H5N9)) were described previously [34]. These virus strains were propagated in embryonated chicken eggs. Allantoic fluids containing the viruses were collected and purified as described previously [34] prior to their use in subsequent experiments. Briefly, allantoic fluids were precleared by centrifugation at 3300× *g* for 20 min and then passed through a syringe filter (0.45 μm pore size). Finally, the virus in the allantoic fluid was purified by ultracentrifugation (112,500× *g* for 2 h) on PBS (without calcium/magnesium) (PBS (−)) containing 20% sucrose (w/v). The resulting pellets were suspended in PBS (−), and the titer was measured in focus-forming assays on MDCK cells (results are expressed as the number of focus-forming units (FFU)/mL) [35] following a slightly modified procedure (the detailed method is described in Section 2.4.). All experiments with live avian viruses were conducted at Kyoto Prefectural University of Medicine under Biosafety Level 3+ conditions (as approved by the Ministry of Agriculture, Forestry, and Fisheries, Japan). The MDCK cells were purchased from the Riken BioResource Center Cell Bank (Ibaragi, Japan). The HTEpCs were purchased from PromoCell Corp. (Heidelberg, Germany) (cells were obtained by PromoCell Corp. with informed consent). Immortalized human bronchiolar epithelial cells (SAEC-Ts) were previously described [34].

### 2.3. Reagents

The MDCK cells were cultured in minimum essential medium supplemented with 10% fetal bovine serum (FBS) and standard antibiotics (penicillin (100 units/mL), streptomycin (100 μg/mL), and amphotericin B (250 ng/mL)). The HTEpCs were cultured in Airway Epithelial Cell Growth Medium (AECGM) (PromoCell) according to the manufacturer’s instructions. The SAEC-Ts and HTEpC-Ts were cultured in D/M medium (DMM) which is based on Dulbecco’s modified Eagle’s medium (DMEM) and MCDB153 (1:1); both media were supplemented with growth factors (bovine pituitary extract (30 μg/mL), hydrocortisone (0.5 μg/mL), epidermal growth factor (0.5 ng/mL), epinephrine (0.5 μg/mL), transferrin (10 μg/mL), insulin (5 μg/mL), triiodothyronine (6.5 ng/mL), retinoic acid (0.1 ng/mL), or cholera toxin (0.1 μg/mL)), 5% FBS, and antibiotics (penicillin (100 units/mL), streptomycin (100 μg/mL), and amphotericin B (250 ng/mL)), as described previously [34]. The HTEpCs were also cultured in DMM prior to their use in infection experiments.

### 2.4. Focus-Forming Assay to Measure Infectious Titers

The MDCK cells, in 96 well plates, were washed three times with PBS (supplemented with calcium/magnesium) (PBS (+)) and then inoculated for 1 h at 37 °C with sample fluid containing virions. After that, the virus inoculum was removed and the cells were washed three times with PBS (+) and overlaid with 1% methylcellulose in minimum essential medium supplemented with 0.2% bovine serum albumin and standard antibiotics (penicillin (100 units/mL), streptomycin (100 μg/mL), and amphotericin B (250 ng/mL)). At 16 h post-infection, the cells were fixed with 4% paraformaldehyde in PBS (−). After washing three times with PBS (−), the cells were stained, as described in Section 2.8, to detect viral antigens. The titers of individual samples (FFU/mL) were determined by counting the number of fluorescent foci in the well under a fluorescence microscope fitted with filters to detect an Alexa Fluor 488-conjugated secondary antibody (see also Section 2.8.).

### 2.5. Establishment of HTEpC-Derived Cell Clones

The HTEpCs were immortalized by transformation with the SV40 large T-antigen gene as described previously [34]. Briefly, the packaging cell line GP2-293 (Takara Bio, Shiga, Japan) was grown in DMEM supplemented with 10% FCS and standard antibiotics. Next, GP2-293 cells in 10 cm dishes were transfected with pVSV-G and pLNCX2 (Takara Bio) using polyethylenimine (Polysciences, Warrington, PA); pLNCX2 contains the gene encoding the SV40 large T-antigen. The medium was replaced at 24 h post-transfection. At 72 h post-transfection, the supernatant containing the retrovirus was collected, passed through a syringe filter (0.45 μm pore size), and purified by ultracentrifugation (112,500× *g* for 2 h) on PBS (−) containing 20% sucrose (w/v). The resulting pellets were suspended in PBS (−). Next, a monolayer of primary HTEpCs was exposed to medium (AECGM supplemented with polybrene (8 μg/mL)) containing the recombinant retrovirus harboring the SV40 large T-antigen gene. After 24 h, the medium was replaced with fresh medium. After another 48 h, G-418 sulfate (100 μg/mL) was added to the medium to isolate the immortalized cells. After 2 weeks of culturing the immortalized cells with G-418 sulfate, single-cell clones were isolated using Scienceware® cloning discs (Merck, Darmstadt, Germany) or isolated by limiting dilution in a 96 well microplate to establish HTEpC-T clones. The HTEpC-T clones were then cultured in DMM prior to their use in experiments. 

### 2.6. Generation of Recombinant H5N3 Viruses

Recombinant viruses were generated using a reverse genetics system based on previously described methods [36,37,38]. Briefly, a pPOLI plasmid [37] containing seven Dk/HK (H5N3) genes (PB2, LC042024; PB1, LC042025; PA, LC042026; NP, LC042028; NA, LC042029; M, LC042030; and NS, LC042031), along with either a virulent hemagglutinin (HA) gene (the Dk/HK (H5N3) (LC042027) gene harboring multiple basic amino acids within its cleavage site), a pPOLI plasmid containing the H5N1 HA genome of Cw/Ky (H5N1) (AB189053), Ck/Eg (H5N1) (AB465592), an A/duck/Egypt/D1Br12/2007 (H5N1) (AB497012), or the genome of the other human isolates described below was transfected into 293T cells, together with pCAGGS expression plasmids [36] encoding WSN PA, PB1, PB2, and NP (which had been co-cultured with CEFs at a ratio of 7:3). The aforementioned seven genome segments of Dk/HK (H5N3) and the HA genome of Cw/Ky (H5N1), Ck/Eg (H5N1), or A/duck/Egypt/D1Br12/2007 (H5N1) were constructed by RT-PCR as described previously [34]. The virulent HA sequence of Dk/HK (H5N3) was constructed by exchanging single basic amino acids within the HA cleavage site for multiple basic amino acids (i.e., N’-TR-C’ for N’-RRKKR-C’) as described previously [39]. The HA sequences of A/Thailand/Kan353/04 (H5N1) (Thailand (H5N1)) (EF541411), A/Indonesia/5/05 (H5N1) (Indonesia (H5N1)) (CY116646), and A/Shanghai/1/06 (H5N1) (Shanghai (H5N1)) (AB462295) were constructed by PCR using overlapping deoxyoligonucleotides corresponding to the published sequence of the HA open reading frame as described previously [34]. The full-length HA sequences of A/chicken/Egypt/ZU30/2016 (H5N1) (KY029058) were prepared by oligonucleotide synthesis using disclosed open reading frame sequences. Non-coding regions of A/chicken/Egypt/ZU30/2016 (H5N1) were derived from that of a related H5N1 strain (A/Egypt/N04915/2014 NIBRG-306 (H5N1) (obtained from GISAID database)), which harbors a gene sequence that shares a higher degree of homology with A/chicken/Egypt/ZU30/2016 (H5N1). Each plasmid was introduced into cells, and acetylated trypsin (5 μg/mL; Merck) was added to the plates at 1 and 4 days post-transfection. At 7 days post-transfection, the culture supernatants were collected and injected into 9 day old chicken eggs. The allantoic fluid was collected at 3 days post-injection and purified as described above, followed by titration on MDCK cells to measure the number of focus-forming units/mL. All recombinant Dk/HK (H5N3) viruses (containing the virulent HA and H5N1 HA genomes) were confirmed by sequencing.

### 2.7. Viral Infection of Cells

The HTEpC-Ts, SAEC-Ts, and primary HTEpCs were cultured in 96 well plates (2.0 × 10^4^ to 3.0 × 10^4^ cells/well), washed twice with PBS (+), and infected with viruses at a multiplicity of infection (m.o.i.) of 10, 1, and 0.1. The m.o.i. was calculated on the basis of the cell number and the titer of the viruses used (the virus titer was determined in focus-forming assays on MDCK cells as described in Section 2.4). The cells were then incubated at 37 °C for 1 h with virus suspended in PBS (+). The viral solution was removed, and the cells were washed twice with PBS (+). The HTEpC-Ts and SAEC-Ts were cultured in DMM containing 5% FBS. The HTEpCs were overlaid with 1% methylcellulose in DMM containing 5% FBS. Virus-infected cells were subjected to immunofluorescence analysis to detect viral antigens.

### 2.8. Immunofluorescence Analysis

Cells were cultured in 96 well plates (2.0 × 10^4^ to 3.0 × 10^4^ cells/well). At 16 h post-infection with the virus, cells were fixed for 30 min at room temperature with PBS (−) containing 4% paraformaldehyde/0.1% Triton X-100, followed by washing three times with PBS (−). Viral antigens were detected by staining the cells with a rabbit polyclonal antibody raised against A/duck/Hong Kong/342/78 (H5N2) (1:1000 dilution in PBS (−)/1% bovine serum albumin) which recognizes the NP and M1 proteins of both avian and human influenza strains. Binding of the primary antibody to viral proteins was detected by an Alexa Fluor 488-conjugated secondary antibody (Thermo Fisher Scientific, Waltham, MA, USA) diluted 1:500 in PBS (−). Cell nuclei were counterstained with Hoechst 33342 (Merck). An IN Cell Analyzer 2200 (GE Healthcare, Chicago, IL, USA) apparatus was used to determine the percentage of each HTEpC-T/SAEC-T clone and primary HTEpC infected with the virus (presented as “% infectivity” in the figures). Images of infected cells were acquired by the IN Cell Analyzer 2200, and the number of antigen-positive cells and cell nuclei visible in the same field was calculated (the photographs comprised 16 different visual fields analyzed at the same time in each experiment using individual viral strains) by IN Cell Developer Toolbox software (GE Healthcare). The percentage of cells infected by the virus was calculated by dividing the number of antigen-positive cells by the total number of nuclei in the same field (×100). The number of cells counted per test well was >500 (at an m.o.i. of 10), >1000 (at an m.o.i. of 1), and >1500 (at an m.o.i. of 0.1) (the number of countable cells decreased in line with the strength of the cytopathic effects against infected cells). The results are expressed as the mean ± SD of at least three independent cultured wells. 

### 2.9. Assessment of Sialic Acid (SA) Expression by Flow Cytometry

The HTEpC-T monolayers were detached from dishes by exposure to 0.025% trypsin/EDTA and then fixed for 30 min at 4 °C with 4% paraformaldehyde. After washing twice with PBS (−) containing 10 mM glycine and once with PBS (−), the cells were blocked for 1 h at 4 °C with PBS (−) containing 1% bovine serum albumin. After washing once with PBS (−), the cells were incubated for 1 h at 4 °C with 2.5 μg/mL *Sambucus nigra* (SNA)-FITC (Vector Laboratories) in PBS (−) containing 0.1% bovine serum albumin to detect sialic acid α2,6-galactose (SAα2,6Gal) moieties or with 2.5 μg/mL *Maackia amurensis* (MAA)-I-FITC (Vector Laboratories) in PBS (−) containing 0.1% bovine serum albumin to detect SAα2,3Gal (Siaα2-3Galβ(1-4) GlcNAc) moieties. In addition, the cells were incubated with 2.5 μg/mL biotinylated MAA-II (Vector Laboratories, Burlingame, CA) in PBS (−) containing 0.1% bovine serum albumin to detect SAα2,3Gal (SAα2,3-Galβ(1-3) GalNAc) moieties, followed by washing three times with PBS (−) and blocking for 1 h at 4 °C with PBS (−) containing 1% bovine serum albumin. After washing once with PBS (−), the cells were incubated with streptavidin-FITC (1 h at 4 °C) (Vector Laboratories). Finally, after washing each batch of cells three times with PBS (−), 10,000 events were acquired by a flow cytometer (FACSCalibur; BD Biosciences, Franklin Lakes, NJ, USA) to measure the fluorescence intensity. The data were analyzed using CellQuest software (BD Biosciences). The MDCK cells were treated or mock-treated for 4 h at 37 °C with *Arthrobacter ureafaciens* sialidase (100 milliunits/mL; Nacalai Tesque, Kyoto, Japan) prepared in PBS (+) (pH 6.8) before lectins were stained using MAA-I, MAA-II, and SNA.

### 2.10. Western Blot Analysis of Virus Binding to HTEpC-T and SAEC-T Clones

Viruses were allowed to bind to established human cell clones cultured in 12 well plates (2.0 × 10^5^ cells/well). Briefly, cells were washed twice with PBS (+), inoculated with viruses at an m.o.i. of 10, and then incubated at 4 °C for 1 h to prevent endocytosis. The cells were then washed five times with ice-cold PBS (+) and lysed in PBS (−) containing 2% SDS. The protein concentration of the resulting cell lysates was measured using the bicinchoninic acid protein quantification kit (Thermo Fisher Scientific). Next, 10 μg of protein per lane was subjected to electrophoresis in a 10% SDS-polyacrylamide gel. The proteins were then transferred to PVDF membranes and blocked overnight at 4 °C in PBS (−) containing 5% non-fat milk and 0.1% Tween 20. The membrane was then exposed for 1 h at room temperature to the aforementioned rabbit polyclonal antivirus antibody (diluted 1:1000 in PBS (−) containing 5% non-fat milk and 0.1% Tween 20), followed by horseradish peroxidase-conjugated donkey anti-rabbit IgG (diluted 1:10,000 in PBS (−) containing 0.1% Tween 20; Jackson ImmunoResearch, West Grove, PA, USA). Between steps, the membranes were washed three times with PBS (−) containing 0.1% Tween 20. Reactive bands were visualized using a chemiluminescence system (Thermo Fisher Scientific) and Fuji XR film.

### 2.11. Evaluation of the Endosomal pH

The pH within the endosomal compartments of the HTEpC-T (LA-9) and SAEC-T (1A5) clones was measured by flow cytometry analysis as described previously [34]. Briefly, HTEpC-T and SAEC-T clones were cultured in 6 cm dishes and then loaded for 10 min at 37 °C with LysoSensor Green DND-189 (1 μm; Thermo Fisher Scientific) prepared in PBS (+). The cells were washed twice with PBS (+) and then cultured in DMM containing 5% FBS for at least 0.5 h to allow the dye to accumulate within acidic vesicles. After staining with LysoSensor Green DND-189, the cells were carefully removed from the culture plates by trypsinization. The fluorescence intensity of 10,000 events was measured using a FACSCalibur flow cytometer (BD Biosciences), and the data were analyzed using CellQuest software (BD Biosciences).

### 2.12. Virus Infection Inhibition Assay

The effect of increasing the endosomal pH on virus infection was examined by incubating HTEpC-T (LA-9) and SAEC-T (1A5) clones for 2 h in DMM containing 5% FBS plus bafilomycinA1 (BafA1) (1.56, 3.125, 6.25, 12.5, or 25 nM; Merck) prior to virus infection (BafA1 increases endosomal pH). After two washes with PBS (+), cells were infected with Cw/Ky (H5N1). The percentage of infected LA-9 and 1A5 cells was determined using an IN Cell Analyzer 2200 (GE Healthcare) after immunostaining at 12 h post-infection as described above.

### 2.13. Production of Progeny Viral Particles in HTEpCs and HTEpC-T Clones

The HTEpCs were infected (in triplicate) with viruses at an m.o.i. of 0.1. The cells were then incubated at 37 °C for 1 h with virus suspended in PBS (+). The viral solution was removed, and the cells were washed twice with PBS (+). The HTEpCs were cultured in DMM containing 0.2% bovine serum albumin and trypsin (1 μg/mL). At the indicated times post-infection, viral RNA (vRNA) titers in the cell culture supernatants of HTEpCs were measured by quantitative real-time RT-PCR as described below. Infectious viral titers in the cell culture supernatant of both HTEpCs and the HTEpC-T clone were also determined in focus-forming assays on MDCK cells as described in Section 2.4.

### 2.14. Quantitative Real-Time RT-PCR 

Progeny vRNA was extracted from the culture supernatant of infected cells using a QIAamp Viral RNA Mini Kit (Qiagen, Hilden, Germany) and measured by quantitative real-time RT-PCR (THUNDERBIRD® Probe One-step qRT-PCR Kit, Toyobo, Osaka, Japan) using primers targeting the M gene as described previously [40]. The primer sequences were as follows: 5’-CCMAGGTCGAAACGTAYGTTCTCTCTATC-3’ (forward), 5’-TGACAGRATYGGTCTTGTCTTTAGCCAYTCCA-3’ (reverse). A TaqMan probe, which is an oligonucleotide with a fluorescent reporter dye attached to the 5’ end and a non-fluorescent quencher (NFQ) attached to the 3’ end, was used for the assay. The oligonucleotide sequence was FAM-ATYTCGGCTTTGAGGGGGCCTG-MGB-NFQ. The sequences of the primers and TaqMan probes were derived from “The Diagnosis Manual for Influenza Viruses, 3rd Edition” by the National Institute of Infectious Disease in Japan. We conducted one-step real-time RT-PCR using the CFX96 Real-Time PCR System (Bio-Rad, Hercules, CA). The cycling conditions were as follows: a reverse transcription step at 50 °C for 10 min prior to an initial denaturation step at 95 °C for 1 min, followed by amplification for 40 cycles (denaturation at 95 °C for 15 s and annealing/extension at 60 °C for 45 s).

### 2.15. Statistical Analysis

All data are expressed as the mean and standard deviation of at least three determinations per experimental condition. Student’s *t*-tests were used for statistical analysis. One-way analysis of variance (ANOVA) was used, followed by Dunnett’s post-hoc test for multiple comparisons. A *p*-value < 0.05 or < 0.01 was considered significant. Statistical analysis was performed using GraphPad Prism Version 8 software (GraphPad Software Inc.).

## 3. Results

### 3.1. Establishment of Human HTEpC-T Clones

To examine the mechanism(s) underlying the susceptibility of cells in the human conducting respiratory tract to AIV infection, we generated cell clones by transforming primary HTEpC with SV40 large T-antigen and then established HTEpC-Ts after cell cloning. We isolated seven HTEpC-T clones, although one of these died out within ten passages. The other six HTEpC-T clones were sub-cultured successfully for ten passages and stably expressed the SV40 large T-antigen (Supplementary Materials Figure S1). The morphology of each cell clone was similar to that of the parent HTEpCs, although the size and shape of the clones (especially LD-2G3) were slightly different from those of the primary cells (Figure S1).

### 3.2. Susceptibility of HTEpC-Ts to Infection by AIVs

**Figure 1:**
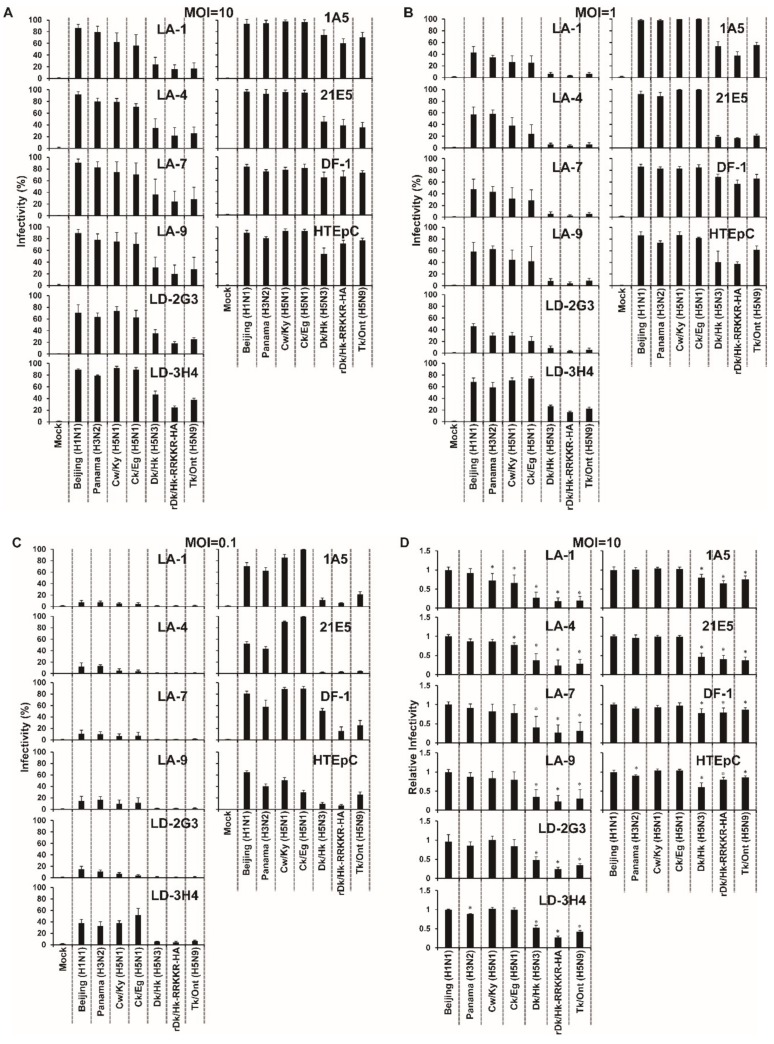

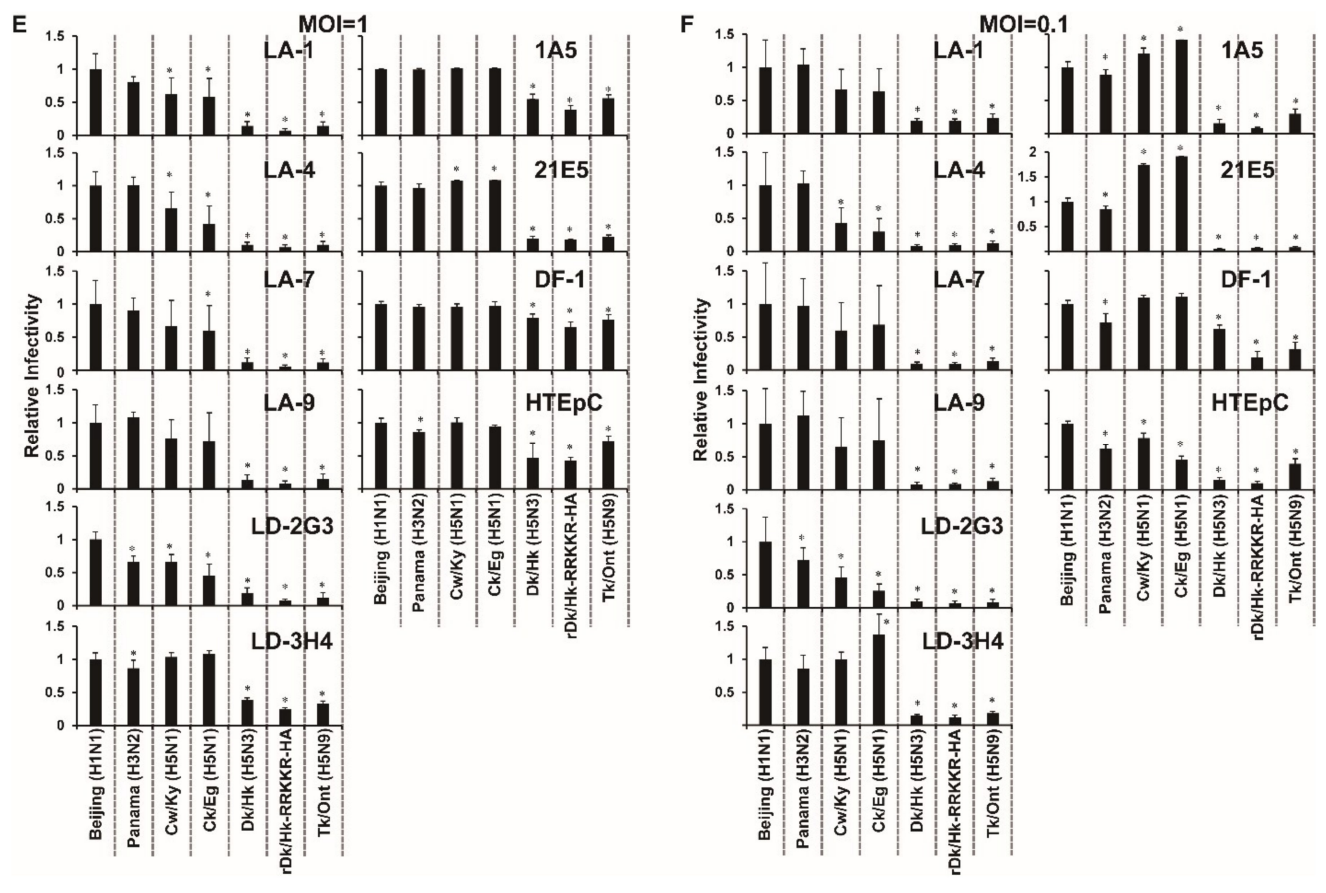
Susceptibility of human tracheal epithelial cell clones to infection by different viruses. (**A**–**C**) HTEpC-T clones were infected with human influenza viruses Beijing (H1N1) and Panama (H3N2); H5N1 (Cw/Ky (H5N1) and Ck/Eg (H5N1)); and previously circulating (non-zoonotic) AIVs Dk/Hk (H5N3), rDk/Hk-RRKKR-HA, and Tk/Ont (H5N9). All cells were infected at an m.o.i. of 10, 1, and 0.1. Viral infectivity was determined by calculating the percentage of antigen-positive HTEpC-T clones after immunostaining at 16 h post-infection. The susceptibility to infection of small airway epithelial cell (SAEC)-T clones (1A5 and 21E5), which were derived from a region different from that of the tracheal tract, chicken-derived cells (DF-1), and primary HTEpCs, was compared with that of the HTEpC-T clones. Data are expressed as the mean ± SD of 11 (LD-2G3 and LD-3H4) or 10 (other cell clones) independent results. (**D**–**F**) Relative cell susceptibility to infection was normalized using the infectivity of Beijing (H1N1). Asterisks indicate that the infectivity of each virus was significantly different from that of Beijing (H1N1) within the same graph. A *p*-value < 0.01 (asterisk) was considered significant (one-way ANOVA followed by Dunnett’s multiple comparisons post-hoc test).

To examine the susceptibility of each HTEpC-T clone to infection by AIVs, we infected them with highly pathogenic H5N1 viruses Cw/Ky (H5N1) and Ck/Eg (H5N1) or with previously circulating (non-zoonotic) AIVs Dk/Hk (H5N3) and Tk/Ont (H5N9). Human influenza viruses (Beijing (H1N1) and Panama (H3N2)) were used as controls. After infection by avian or human viruses, HTEpC-Ts expressed viral antigens (Figure S2); however, the infectivity (the ratio of antigen-expressing cells to total cells in the same field) was different among the tested viruses (Figure 1A–C). The HTEpC-Ts showed differing susceptibility to infection by non-zoonotic AIVs and H5N1/human influenza viruses; however, the overall pattern was similar for all clones. All HTEpC-T clones were highly susceptible to infection by H5N1/human influenza viruses (although the degree of susceptibility to H5N1 varied from clone to clone), but they were significantly less susceptible to infection by non-zoonotic AIVs (H5N3 and H5N9) than to infection by Beijing (H1N1) under all conditions (m.o.i. of 0.1, 1, and 10) (Figure 1D–F). In addition, to examine whether the results in HTEpC-Ts can be replicated in parental HTEpCs, which are primary cultured cells, we infected the latter with human influenza, H5N1, and non-zoonotic AIVs (Figure 1A–C). Primary HTEpCs were highly susceptible to infection by two H5N1 strains, as well as human influenza viruses, at an m.o.i. of 1 and 10 (there was no statistical significance between Beijing (H1N1) and Cw/Ky (H5N1) or Ck/Eg (H5N1)), despite the statistically lower susceptibility of HTEpCs to infection by these two H5N1 strains relative to infection by Beijing (H1N1) at an m.o.i. of 0.1 (Figure 1D–F). However, primary HTEpCs were significantly less susceptible to infection by non-zoonotic AIVs (H5N3 and H5N9) than to infection by Beijing (H1N1) as were HTEpC-T clones under all infectious conditions (m.o.i. of 0.1, 1, and 10) (Figure 1D–F). Notably, H5N1 viruses showed higher infectivity than non-zoonotic AIVs, even in chicken-derived cells (DF-1) known to be broadly susceptible to AIVs [41] (although DF-1 cells were markedly susceptible to infection by non-zoonotic AIVs) (Figure 1A–F).

In addition, to examine differences within different regions of the human airway, we compared the susceptibility of HTEpC-Ts with that of other clones (i.e., 1A5 and 21E5 cells derived from primary human bronchiolar epithelial cells). Hence, 1A5 and 21E5 cells were also transformed by the SV40 large T-antigen and named SAEC-Ts; the susceptibility of 1A5 and 21E5 cells to non-zoonotic AIVs is “adequate” and “inadequate”, respectively [34], although both clones were derived from the same donor. As previously reported, 1A5 cells were highly susceptible to infection by H5N1; the infection was similar to (m.o.i. of 10 and 1) or significantly higher (m.o.i. of 0.1) than that by Beijing (H1N1). The cells were also susceptible to non-zoonotic AIVs at an m.o.i. of 10 and 1, although the susceptibility to non-zoonotic AIVs was not as high as that to Beijing (H1N1) (Figure 1A–F) [34]. The 21E5 cells were also susceptible to H5N1 viruses; their susceptibility was similar to (m.o.i. of 10) or significantly higher (m.o.i. of 1 and 0.1) than that to Beijing (H1N1), despite the fact that 1A5 and 21E5 cells differ in their susceptibility to infection by non-zoonotic AIVs (Figure 1A–F). As seen in 1A5 and 21E5 cells, variable susceptibility to non-zoonotic AIVs was observed among the tested cell clones (Figure 1A–F). Notably, the susceptibility of HTEpC-T clones to infection by non-zoonotic AIV Dk/Hk (H5N3) was similar to that of 21E5 cells (Figure 1A–F) under all infectious conditions (m.o.i. of 10, 1, and 0.1) but different from that of 1A5 cells. This suggests that the mechanism(s) that determines the susceptibility of HTEpC-Ts to infection by non-zoonotic AIVs is similar to that in 21E5 cells but different from that in 1A5 cells (Figure 1A–F).

We focused on the observation that all clones showed uniformly highly susceptibility to infection by H5N1 viruses. The presence of multiple basic amino acids within the HA cleavage site correlates with the virulence of highly pathogenic AIVs in both birds and mammals [42,43]. To examine whether differences in this amino acid motif within the HA cleavage sites of Cw/Ky (H5N1) and Dk/Hk (H5N3) contribute to their differing ability to infect HTEpC-Ts and 21E5 cells, we examined the susceptibility of these cell clones to a recombinant H5N3 virus harboring a sequence of multiple basic amino acids from Cw/Ky (H5N1) (N’-RRKKR-C’: corresponding to amino acid position 325–329 in HA, according to H3 numbering [44]) within the HA cleavage site (rDk/Hk-RRKKR-HA). As shown in Figure 1, both rDk/Hk-RRKKR-HA and wild-type Dk/Hk (H5N3) infected HTEpC-Ts and 21E5 cells to a similar extent, suggesting that the multiple basic amino acid sequences within H5N1 HA are not responsible for its ability to infect these cell lines.

**Figure 2:**
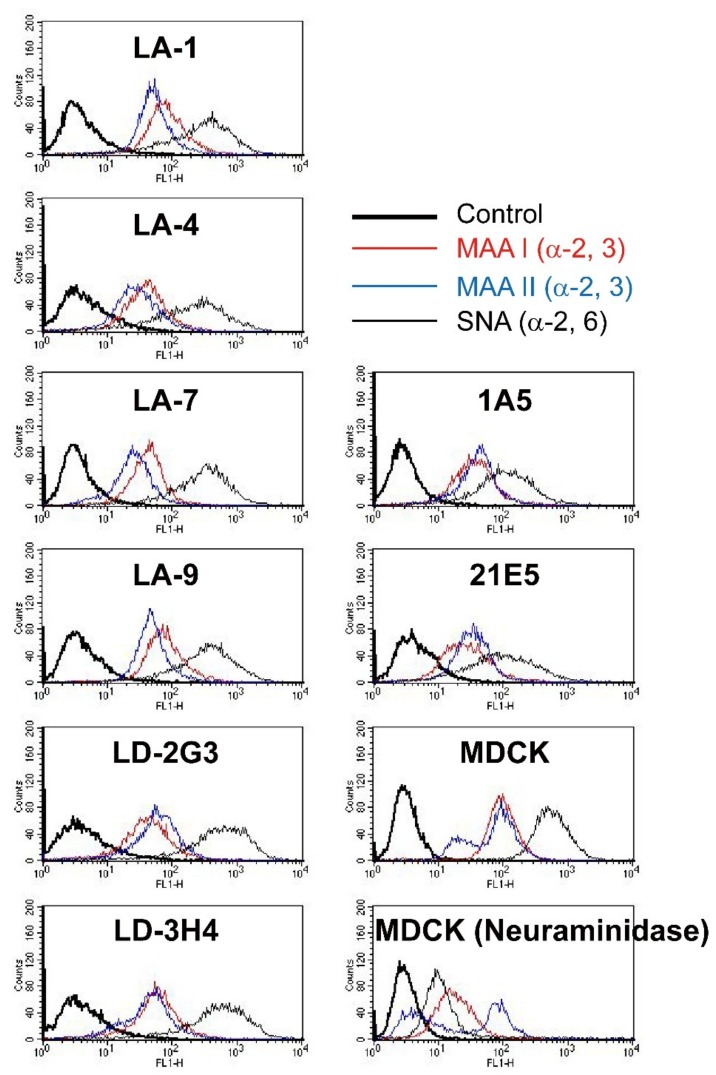
Expression of virus receptors by human tracheal epithelial cell clones. The expression of sialic acid (SA) receptors on the surface of the HTEpC-T clones was analyzed by flow cytometry. α2,3SAGal and α2,6SAGal receptors were detected by *Maackia amurensis* (MAA) and *Sambucus nigra* (SNA) lectins, respectively. α2,3SAGal residues are shown in red (when detected by MAA-I) or blue (when detected by MAA-II). α2,6SAGal is shown as a black line. Control cells (without lectin) are represented by the black bold line. SAEC-T clones (1A5 and 21E5), which were derived from primary human bronchiolar epithelial cells, were also examined, and receptor expression patterns were compared with those of HTEpC-T clones. Madin–Darby canine kidney (MDCK) cells (virus-susceptible controls) express both α2,3SA and α2,6SA receptors. MDCK cells were first treated with neuraminidase to confirm the reliability of lectin staining.

To assess the cell characteristics associated with susceptibility to viral infection, we also examined the expression of viral receptors SAα2,3Gal and SAα2,6Gal on HTEpC-Ts by staining cells with MAA-I/II and SNA. Both SAα2,3Gal and SAα2,6Gal were expressed by all HTEpC-T clones as well as SAEC-Ts (1A5 and 21E5 cells) and MDCK cells (virus-susceptible controls) (Figure 2).

### 3.3. Binding of Viruses to HTEpC-T Clones

**Figure 3:**
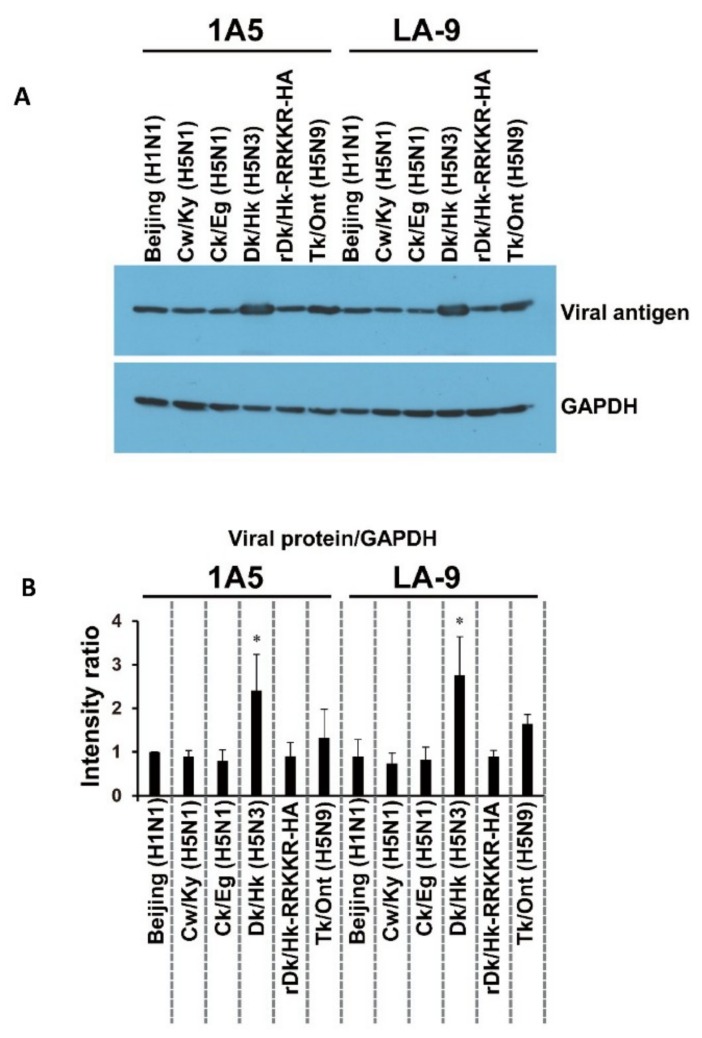
Binding of viruses to human small airway epithelial cell clones and to human tracheal epithelial cell clones. (**A**) The binding of viruses to human SAEC-T clone 1A5 and HTEpC-T clone LA-9 was analyzed by Western blotting. Clones 1A5 and LA-9 were inoculated with human influenza strain Beijing (H1N1), H5N1 (Cw/Ky (H5N1) or Ck/Eg (H5N1)), or previously circulating (non-zoonotic) Avian influenza viruses (Dk/Hk (H5N3), rDk/Hk-RRKKR-HA, or Tk/Ont (H5N9)) at an m.o.i. of 10. Cells were inoculated with viruses for 1 h at 4 °C and harvested immediately. Viral protein was detected with an anti-H5N2 polyclonal antibody. GAPDH was used as a control. The intensity of the bands representing each viral protein and GAPDH was measured using ImageJ. Representative images of the viral protein (M1) and GAPDH are shown. Uncropped images are shown in Figure S3. (**B**) Summary of the relative intensities of bands corresponding to the viral protein (M1) and GAPDH (shown in (**A**)). The intensities of the bands representing each viral protein were normalized using those of Beijing (H1N1) viral proteins in 1A5 cells; the intensities of the bands representing GAPDH were normalized using those of 1A5 cells infected with Beijing (H1N1). The intensities of Beijing (H1N1) viral proteins/GAPDH in 1A5 cells were set to 1. Data are expressed as the mean ± SD of three independent results. Asterisks indicate that the relative intensity of the bands representing each viral protein was significantly higher than that representing Beijing (H1N1) in 1A5 cells. A *p*-value < 0.01 (asterisk) was considered significant (one-way ANOVA followed by Dunnett’s multiple comparisons post-hoc test).

Next, we examined the mechanism by which AIVs infect HTEpC-Ts by examining the binding of H5N1, non-zoonotic AIVs, and human influenza viruses (used as a control) to the cells. Representative HTEpC-T (LA-9) and SAEC-T (1A5) clones (1A5 is adequately susceptible to AIVs [34], so it was used as a control) were tested. Briefly, 2.0 × 10^5^ cells were inoculated with the virus at an m.o.i. of 10, lysed, and then immunoblotted with an antibody specific to the viral antigen (see Section 2.). We found no marked difference in the number of virions bound to 1A5 or LA-9 cells. Specifically, the number of virions from each strain that bound to each clone was similar, although the number of Dk/Hk (H5N3) virions bound to 1A5 and LA-9 cells was significantly higher than the number of Beijing (H1N1) virions (Figure 3A,B). Notably, non-zoonotic H5 viruses (Dk/Hk (H5N3), rDk/Hk-RRKKR-HA, and Tk/Ont (H5N9)), which do not usually infect humans, bound to HTEpC-T clone LA-9.

### 3.4. Comparison of the Endosomal pH in HTEpC-T and SAEC-T Clones

**Figure 4:**
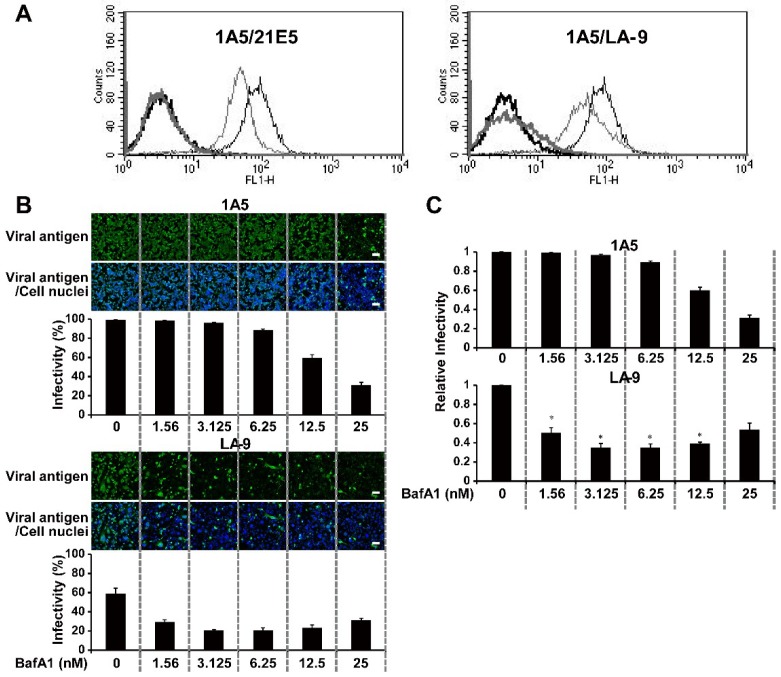
pH levels in acidic compartments within human small airway epithelial cell clones and human tracheal epithelial cell clones. (**A**) Human SAEC-T clone 1A5 and HTEpC-T clone LA-9 were stained with LysoSensor Green DND-189 and then analyzed by flow cytometry. The profiles of the stained cells are shown as black (1A5) and gray (21E5 and LA-9) lines. The profile of 1A5 cells is the same in the left and right panels. Control cells (no staining) are denoted by bold lines. (**B**) Human SAEC-T clone 1A5 and HTEpC-T clone LA-9 were treated with bafilomycinA1 (BafA1; 0–25 nM) for 2 h and then infected with Cw/Ky (H5N1) at an m.o.i. of 10. Twelve hours later, infectivity was determined by calculating the percentage of antigen-positive cells (as described in Figure 1). Representative micrographs of antigen-positive cells treated with BafA1 are shown. Cell nuclei were also counted. Viral infectivity was determined as described in Figure 1. Data are expressed as the mean ± SD of three independent results. Scale bars, 100 μm. (**C**) Summary of relative infectivity in panel (**B**). The infectivity in the absence of reagent was set to 1. Asterisks indicate an infectivity ratio that was significantly lower than that for 1A5 cells at the same concentration of reagent. A *p*-value < 0.01 (asterisk) was considered significant (Student’s *t*-test).

After cell binding and endocytosis, influenza viruses were trafficked to the late endosomes, in which the acidic milieu induces virus–cell membrane fusion and subsequent release of the viral genome into the cytoplasm [45,46,47]. Because the number of Cw/Ky (H5N1) and Ck/Eg (H5N1) virions bound to the cells was not higher than that of the virions of other viral strains, including non-zoonotic AIVs (Dk/Hk (H5N3) and Tk/Ont (H5N9)), we hypothesized that differences in the pH of late endosomes may determine susceptibility to viral infection. Therefore, we used LysoSensor Green DND-189 (which accumulates in acidic organelles, such as late endosomes and lysosomes, and releases green fluorescence when the pH is low) to examine the endosomal pH of clones LA-9 and 1A5 [48,49,50]. Flow cytometry analysis revealed that the fluorescence intensity in acidic organelles of 1A5 cells was higher than that within those of LA-9 cells (Figure 4A). Collectively, these data suggest that the pH in the late endosomes of LA-9 cells is higher than that in those of 1A5 cells.

### 3.5. Viral Infectivity Is Regulated by Gradual Changes in Endosomal pH

The different endosomal pH values observed for HTEpC-T clone LA-9 and SAEC-T clone 1A5 prompted us to examine the relationship between endosomal pH values and cell susceptibility to infection by AIVs. To do this, we increased the endosomal pH of both LA-9 and 1A5 cells by treatment with BafA1 which increases the endosomal pH by inhibiting vacuolar-type H+-ATPase activity. Even in 1A5 cells, which are highly susceptible to AIVs [34], BafA1 prevented infection by the H5N1 virus in a dose-dependent manner (Figure 4B). Much lower concentrations of BafA1 inhibited viral infection of LA-9 cells (Figure 4B,C), confirming that the endosomal pH of LA-9 cells is higher than that of 1A5 cells.

### 3.6. The Effect of HA Acid Stability on the Susceptibility of HTEpC-Ts to Infection

**Figure 5:**
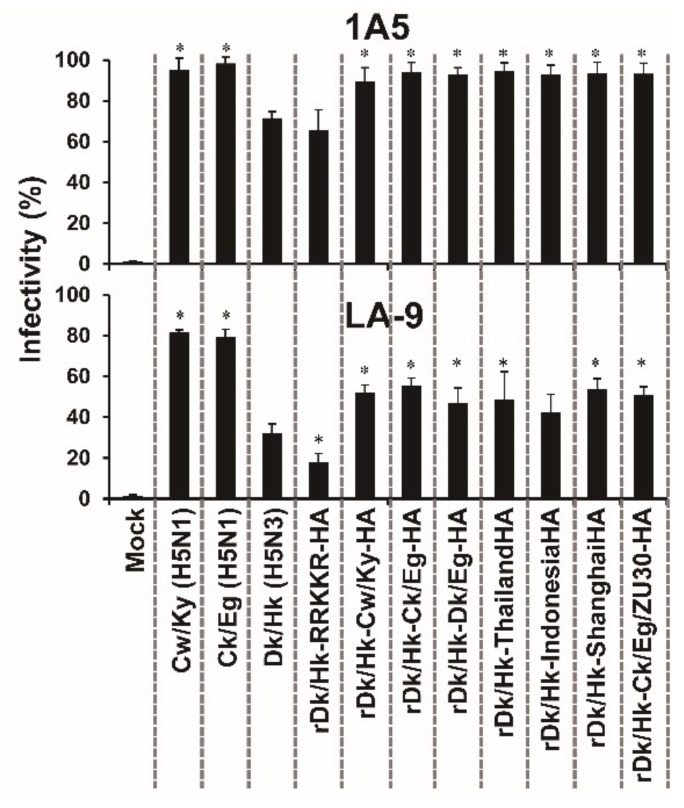
Susceptibility of human small airway epithelial and tracheal epithelial cell clones to infection by recombinant H5N3 viruses harboring the H5N1 HA gene. Human SAEC-T clone 1A5 and HTEpC-T clone LA-9 were infected (m.o.i. = 10) with Dk/Hk (H5N3), rDk/Hk-RRKKR-HA, and recombinant H5N3 (rDk/Hk-Cw/Ky-HA, rDk/Hk-Ck/Eg-HA, rDk/Hk-Dk/Eg-HA rDk/Hk-Thailand-HA, rDk/Hk-Indonesia-HA, rDk/Hk-Shanghai-HA, or rDk/Hk-Ck/Eg/ZU30-HA) viruses containing the HA gene from H5N1 (A/crow/Kyoto/53/04 (clade 2.5), A/chicken/Egypt/CL6/07 (clade 2.2.1), A/duck/Egypt/D1Br12/2007 (clade 2.2.1), A/Thailand/Kan353/04 (clade 1), A/Indonesia/5/05 (clade 2.1.3), A/Shanghai/1/06 (clade 2.3.4), or A/chicken/Egypt/ZU30/2016 (clade 2.2.1.2)). Viral infectivity was determined as described in Figure 1. The H5N1 HA amino acid sequence of A/duck/Egypt/D1Br12/2007 (clade 2.2.1) is identical to that of the human isolate A/Egypt/902786/2006 (clade 2.2.1) (EU146868). Data are expressed as the mean ± SD of eight independent results. Asterisks indicate that the infectivity of each virus was significantly different from that of Dk/Hk (H5N3) within the same graph. A *p*-value < 0.01 (asterisk) was considered significant (one-way ANOVA followed by Dunnett’s multiple comparisons post-hoc test).

Next, we examined viral factor(s) that may affect infectivity. Because the infection of cells requires viral–cell membrane fusion, which is induced by a pH-dependent HA conformational change, we investigated whether the acid stability of the HA molecule affects the susceptibility of HTEpC-Ts to viral infection. Previously, we reported that H5N1 viruses undergo HA-dependent viral–cell membrane fusion at higher pH values than non-zoonotic AIVs [34] (the pH threshold for the HA protein to induce membrane fusion is summarized in Supplementary Materials Table S1). Therefore, we generated recombinant Dk/Hk (H5N3) viruses showing different pH sensitivities to induce membrane fusion and then examined viral infection. Recombinant Dk/Hk (H5N3) harboring H5N1 HA genes from avian isolate Cw/Ky (H5N1) (clade 2.5), Ck/Eg H5N1 (clade 2.2.1), A/duck/Egypt/D1Br12/2007 (H5N1) (clade 2.2.1), or A/chicken/Egypt/ZU30/2016 (H5N1) (clade 2.2.1.2) (defined here as rDk/Hk-Cw/Ky-HA, rDk/Hk-Ck/Eg-HA, rDk/Hk-Dk/Eg-HA, and rDk/Hk-Ck/Eg/ZU30-HA, respectively) or H5N1 HA genes from human isolates A/Thailand/Kan353/04 (clade 1), A/Indonesia/5/05 (clade 2.1.3), or A/Shanghai/1/06 (clade 2.3.4) (defined here as rDk/Hk-ThailandHA, rDk/Hk-Indonesia, and rDk/Hk-ShanghaiHA, respectively) was able to infect LA-9 cells with a mildly acidic endosomal pH environment as did wild-type Cw/Ky (H5N1) and Ck/Eg (H5N1) (Figure 5). The infectivity of recombinant Dk/Hk (H5N3) harboring H5N1 HA genes was significantly higher than that of the parent virus (Dk/Hk (H5N3)) in LA-9 cells. By contrast, recombinant Dk/Hk (H5N3) harboring sequences with multiple basic amino acids (N’-RRKKR-C’) within the HA cleavage site (rDk/Hk-RRKKR-HA) derived from Cw/Ky (H5N1) infected LA-9 cells poorly as did the parent virus Dk/Hk (H5N3). These viruses were able to infect 1A5 cells, which have an acidic endosomal pH, although the infectivity of recombinant Dk/Hk (H5N3) harboring H5N1 HA genes was significantly higher than that of the parent virus Dk/Hk (H5N3) in 1A5 cells (Figure 5).

### 3.7. The Effect of HA Acid Stability on the Susceptibility of Primary HTEpCs to Infection

**Figure 6:**
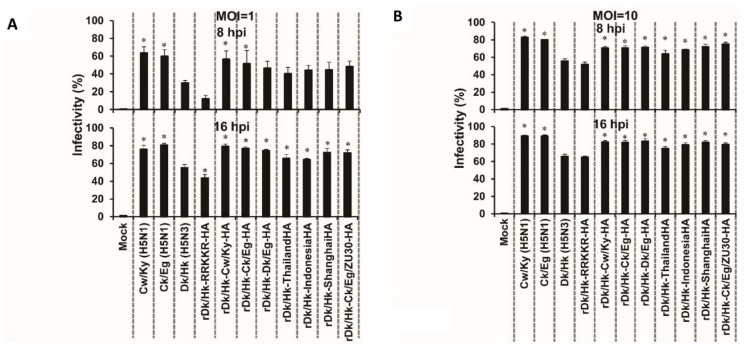
Susceptibility of primary human tracheal epithelial cells to infection by recombinant H5N3 viruses harboring the H5N1 HA gene. Human HTEpCs were infected (8 and 16 hpi at an m.o.i. of 1 (**A**) and 10 (**B**)) with H5N1 (Cw/Ky (H5N1) and Ck/Eg (H5N1)), Dk/Hk (H5N3), rDk/Hk-RRKKR-HA, and recombinant H5N3 containing the HA gene from H5N1 viruses (as described in Figure 5). Data are expressed as the mean ± SD of four independent results. Asterisks indicate that the infectivity of each virus was significantly different from that of Dk/Hk (H5N3) within the same graph. A *p*-value < 0.01 (asterisk) was considered significant (one-way ANOVA followed by Dunnett’s multiple comparisons post-hoc test).

To examine whether the effect of HA acid stability on the susceptibility of HTEpC-Ts can be replicated in HTEpCs, which are primary cultured cells, we infected the latter with recombinant Dk/Hk (H5N3) harboring the H5N1 HA genes described above. The susceptibility of HTEpCs to infection by these AIVs was similar to that of HTEpC-Ts (Figure 5 and Figure 6). The HTEpCs were highly susceptible to infection by H5N1 and recombinant Dk/Hk (H5N3) harboring H5N1 HA genes; however, they were only moderately susceptible to infection by H5N3 and rDk/Hk-RRKKR-HA (as were HTEpC-T clones) (Figure 6A,B). In HTEpCs, the infectivity of recombinant Dk/Hk (H5N3) harboring H5N1 HA genes was significantly higher than that of the parent virus Dk/Hk (H5N3) (Figure 6A,B). By contrast, the susceptibility of primary HTEpCs and HTEpC-T clones to infection by non-zoonotic AIVs Dk/Hk (H5N3) and rDk/Hk-RRKKR-HA showed some differences; primary HTEpCs were more susceptible to infection by Dk/Hk (H5N3) and rDk/Hk-RRKKR-HA than HTEpC-T clones, especially at an m.o.i. of 10 (Figure 1, Figure 5 and Figure 6A,B).

### 3.8. Viral Growth Kinetics in Primary HTEpCs and Their Established Clones

**Figure 7:**
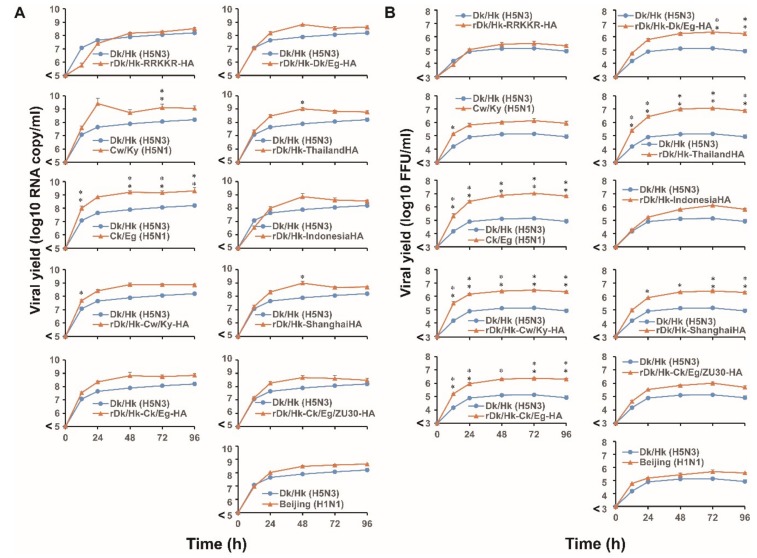

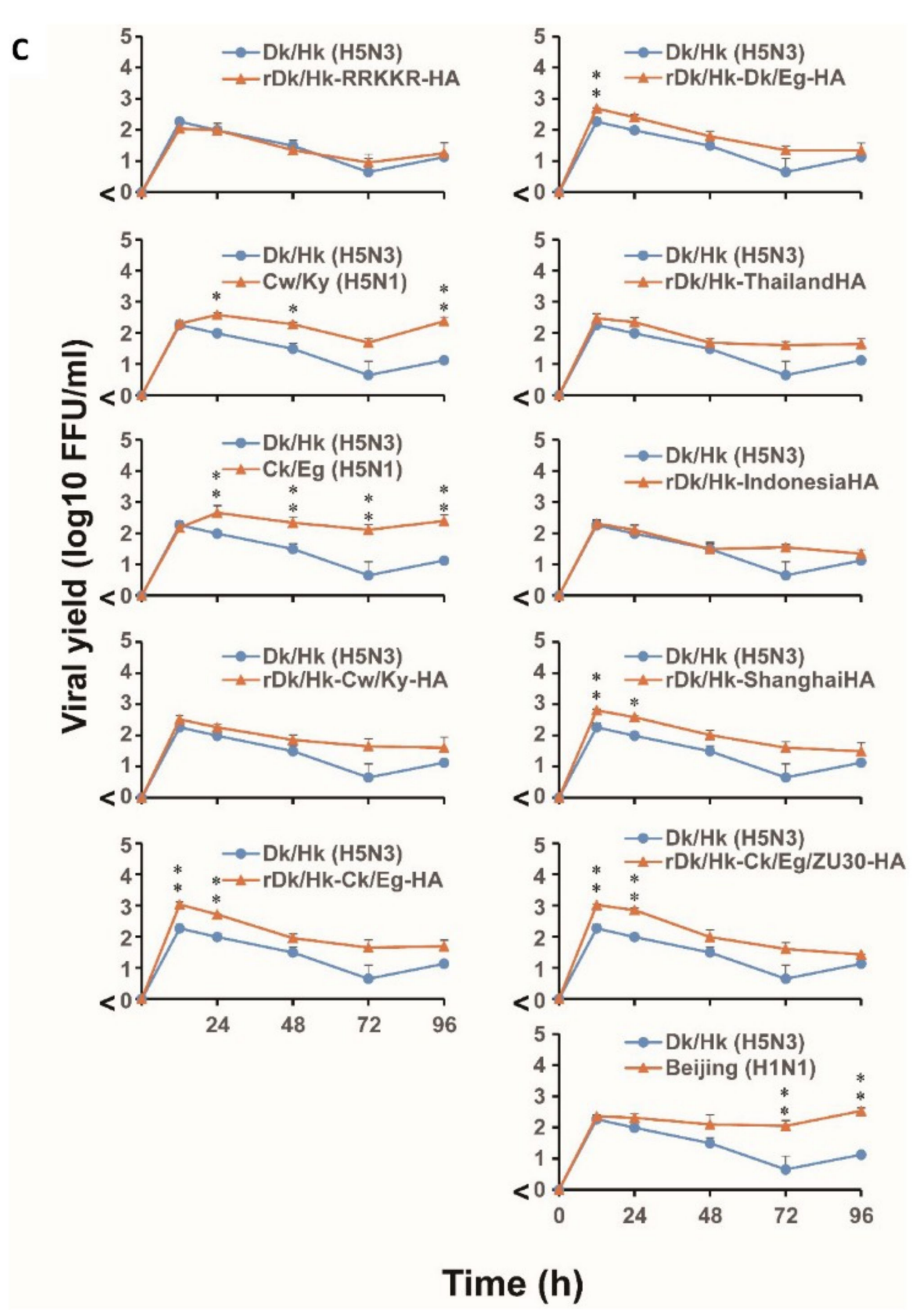
Growth kinetics of avian influenza viruses and their recombinant viruses in primary human tracheal epithelial cells and their established clones. (**A**) HTEpCs were infected with H5N1 (Cw/Ky (H5N1) and Ck/Eg (H5N1)), Dk/Hk (H5N3), rDk/Hk-RRKKR-HA, and recombinant H5N3 containing the HA gene from H5N1 viruses (as described in Figure 5) at an m.o.i. of 0.1 and incubated at 37 °C. At the indicated times post-infection, the amount of progeny vRNA within the culture supernatants was determined by measuring virus titers in quantitative real-time PCR assays. (**B**) The infectious viral titers in infected cells were measured by infecting HTEpCs with AIVs and recombinant viruses, as described in (**A**). At the indicated times post-infection, the infectious titer of progeny virions in the culture supernatants was determined in a focus-forming assay. (**C**) The representative HTEpC-T clone LA-9 was infected with the AIVs described in (**A**) under the same infectious conditions. At the indicated times post-infection, the infectious titer of progeny virions in the culture supernatants was determined in a focus-forming assay. Data are expressed as the mean ± SD of three independent results. Asterisks indicate virus titers that are significantly higher than that of Dk/Hk (H5N3). A *p*-value < 0.05 (single asterisk) or < 0.01 (double asterisk) was considered significant (one-way ANOVA followed by Dunnett’s multiple comparisons post-hoc test).

In the final series of experiments, we examined the possible relationship between susceptibility to infection and viral growth kinetics in both HTEpCs and their established clones. We infected HTEpCs with H5N1, non-zoonotic virus, and its recombinant AIVs at a low m.o.i. (0.1). The replication kinetics of the H5N1 virus and recombinant Dk/Hk (H5N3) harboring H5N1 HA genes were significantly higher than those of Dk/Hk (H5N3), regardless of the H5N1 strain (Figure 7). The kinetics of rDk/Hk-RRKKR-HA were similar to those of the parent virus Dk/Hk (H5N3) (Figure 7A,B). We observed a similar pattern between the copy number and infectious viral titer of virions released from the infected cells, although the copy number was higher than the infectious titer (Figure S4). This result was reflected by the positive correlation between the copy number and infectious viral titer for each recombinant viral strain (Figure S4). We also evaluated viral replication kinetics in LA-9 cells and compared the results for primary HTEpCs with those for HTEpC-T. The viral growth curve obtained for LA-9 cells was similar to that for primary HTEpCs, despite the lower infectious titer in LA-9 cells (Figure 7). The replication kinetics of the H5N1 virus in LA-9 cells were significantly higher than those of Dk/Hk (H5N3). The growth kinetics of recombinant Dk/Hk (H5N3) harboring H5N1 HA genes were also significantly higher than those of the parent virus Dk/Hk (H5N3) in LA-9 cells, although statistical significance was rarely observed for some recombinant viral strains harboring H5N1 HA genes (Figure 7C).

## 4. Discussion

Here, we report four main findings with respect to the mechanism underlying the susceptibility of human tracheal cells to infection by AIVs: (i) HTEpC-Ts showed differing susceptibility to infection by H5N1 and non-zoonotic AIVs; (ii) viral receptors expressed by HTEpC-Ts were able to bind viral particles at similar levels; (iii) the pH in the endosomes of HTEpC-Ts determined their susceptibility to infection by AIVs; and (iv) the H5N1 HA protein, with a higher pH threshold for HA-mediated membrane fusion, broadened the viruses’ tropism for HTEpC-Ts.

Both SAEC-T clone 21E5 and HTEpC-Ts were highly susceptible to infection by human influenza and H5N1 viruses, but they were less susceptible to infection by non-zoonotic AIVs, such as H5N3 and H5N9, regardless of the presence of multiple basic amino acids in the HA cleavage site (Figure 1). The susceptibility of HTEpC-Ts is in agreement with the results of other studies on receptor binding and/or replication of AIVs [51,52,53,54]. For example, van Riel et al. [53,54] showed that AIVs, including H5 subtypes, bound weakly to sections of the human trachea and bronchus but moderately to bronchioles. By contrast, Matrosovich et al. [51] confirmed that AIV strain A/mallard/Alberta/119/98 (H1N1) infects differentiated nasal and tracheobronchial cells of the human airway. Furthermore, Nicholls et al. [52] used ex vivo tissue culture models to demonstrate the presence of novel viral antigens in the human upper respiratory tract (i.e., nasopharynx, adenoid, and tonsil) with A/Vietnam/3046/04 (H5N1) at 24 h post-infection. Moreover, they showed that the number of progeny virions in both nasopharyngeal biopsy and primary nasopharyngeal epithelial cells infected with the same virus strain increased over time. The results reported herein, together with those in the studies described above, suggest that the human upper respiratory or conducting airways (e.g., the trachea) may be susceptible to AIVs, although the degree of infectivity may vary. By contrast, we found here that individual HTEpC-T clones show slightly different susceptibility to infection, even by the same viral strain (Figure 1). This variable susceptibility among HTEpC-Ts could be due to the different cellular origins (e.g., ciliated epithelial cells, goblet cells, Clara cells, and basal cells) or a different differentiation stages within the same cell. The variability observed in HTEpC-T clones is likely to correlate with the different morphology of HTEpC-Ts (i.e., morphological differences between LD-2G4 and other cell clones) (Figure S1).

The patterns of viral receptor expression in the human airways could be associated with differences in susceptibility within different regions of the human airway. Some studies have reported that the avian receptor SAα2,3Gal (stained by MAA-II) is expressed mainly in the terminal part of the human LRT [17,55], suggesting that human infection by AIVs could be restricted to bronchioles and alveoli. However, the SAα2,3Gal avian receptor can also be stained by another lectin, MAA-I. In this study, staining HTEpC-Ts with lectins revealed abundant expression of SAα2,3Gal (stained by either MAA-I or MAA-II) and SAα2,6Gal residues (stained by SNA), which are recognized by avian and human influenza viruses, respectively (Figure 2). However, the pattern of SA expression by HTEpC-Ts does not correspond completely with the pattern in human respiratory tissue (in vivo). Generally, SAα2,6Gal is expressed in the upper to lower airway; by contrast, the analysis of the expression of glycanic structures (i.e., the sub-terminal residues of SAα2-3 oligosaccharides) suggests that expression of SAα2,3Gal differs according to variations in the sub-terminal SAα2-3 oligosaccharide residues [52,56,57,58,59]. For example, SAα2,3-Galβ(1-4)GlcNAc, which is an N-linked glycan recognized by MAA-I, is likely to be expressed throughout the whole respiratory tract (although expression in the tracheal region has not been well analyzed), whereas that of SAα2,3-Galβ(1-3)GalNAc, which is an O-linked glycan recognized by MAA-II, is detected mainly in the bronchioles and alveoli [17,55,57,59]. As shown by both Matrosovich et al. [51] and Nicholls et al. [52], as well as the data presented herein, one reason that AIVs infect epithelial cells lining the upper and conducting airways is their expression of SAα2,3-Galβ(1-4)GlcNAc which is recognized by MAA-I and expressed broadly throughout the human respiratory tract. The presence of SAα2,3-Galβ(1-4)GlcNAc suggests that AIVs may bind to and/or infect epithelial cells lining the human conducting airways in vivo and in vitro; however, our finding that AIVs infect HTEpC-Ts may also be due to the fact that the HTEpC-T clones express high levels of the avian receptor SAα2,3-Galβ(1-3)GalNAc, which is rarely expressed in the human trachea in vivo [17,55], as well as SAα2,3-Galβ(1-4)GlcNAc. This unexpected pattern of viral receptor expression may be caused by the conditions used to culture HTEpC-Ts. Unfortunately, the characteristics of cells in liquid culture do not always mirror those of their in vivo counterparts, because the differentiation levels are different. Usually, traditional culture systems do not enable complete cell differentiation; indeed, full differentiation requires an air–liquid interface (ALI) culture system which allows for the generation of fully differentiated epithelial cells such as ciliated cells, goblet cells, and Clara (club) cells [60,61,62,63]. Therefore, the unexpected (and sufficient) expression of SAα2,3-Galβ(1-3)GalNAc by the HTEpC-Ts examined herein may be due to the lack of differentiation. Another possibility is individual differences in the expression of SAα2,3-Galβ(1-3)GalNAc by the primary epithelial cells from which the HTEpC-Ts were derived. Indeed, one limitation of this study was that the HTEpC-Ts were derived from only one donor. Further studies should examine HTEpC-Ts derived from multiple donors and use both “traditional” and ALI culture systems.

The HTEpC-Ts maintained in liquid culture expressed high levels of SA (SAα2,3Gal and SAα2,6Gal) residues; this explains the high numbers of viral particles bound to the cells in the virus-binding assay (Figure 3). The analysis of cell surfaces showed that H5N1, H5N3, and H5N9 viruses bound similarly to HTEpC-T (LA-9) and SAEC-T (1A5) cells which are marginally and adequately susceptible, respectively, to infection by AIVs. These results suggest that an event(s) arising after viral internalization by endocytosis is responsible for differences in the susceptibility of LA-9 and 1A5 cells to infection by AIVs. After internalization via endocytosis, influenza viruses undergo a low-pH-dependent conformational change in the HA molecule, leading to membrane fusion and subsequent infection [45,46,47]. Previously, we showed that H5N1 viruses harboring an HA gene from a different clade have a higher pH threshold (pH 5.625–5.75) for HA-mediated membrane fusion than non-zoonotic avian H5 viruses such as Dk/Hk (H5N3) and Tk/Ont (H5N9) (pH 5.125–5.375) (see Table S1) [34]. The higher pH threshold of H5N1 viruses may account for the results reported herein, i.e., all HTEpC-T and SAEC-T (1A5 and 21E5) clones were highly susceptible to H5N1. By contrast, differences in the susceptibility of LA-9 and 1A5 cells to infection by Dk/Hk (H5N3) and Tk/Ont (H5N9) were associated with cellular endosomal pH values; H5N3 and H5N9 viruses do not undergo viral–cell membrane fusion until the endosomal pH becomes acidic (pH threshold of HA: pH 5.125–5.375) [34]. This prompted us to compare the endosomal pH values in different clones. Flow cytometry analysis using LysoSensor Green DND-189 revealed that the pH in the late endosomes of LA-9 cells is higher than that in those of 1A5 cells (Figure 4A), which supports our hypothesis that the endosomal pH in individual epithelial cell clones determines their susceptibility to infection by AIVs. The role of different endosomal pH values in 1A5 and LA-9 cells was highlighted by treating cells with BafA1. The infection of LA-9 cells was more sensitive to the alkalinizing effect of BafA1 than that of 1A5 cells (Figure 4B,C); this finding indicates that the pH environment in the endosomes differs considerably among these cells, and it also confirms the data from the flow cytometry experiments using a pH-dependent indicator which showed that the endosomal pH in LA-9 cells was higher than in 1A5 cells. From a physiological point of view, epithelial cells (such as LA-9 cells) with a mildly acidic endosomal pH environment may help to defend tissues from some pathogens including viruses and toxins that can be activated by an acidic environment. Thus, human tracheal epithelia, including cells with a mildly acidic endosomal pH, might protect the conducting airways from invasion, although the ratio of cells with an acidic/mildly acidic endosomal pH in tracheal epithelia is unknown at this moment. Thus, we found that the less acidic endosomal environment within HTEpC-Ts enables them to resist infection by H5N3 and H5N9 viruses. By contrast, HTEpC-Ts may succumb to infection by H5N1 viruses because the higher pH threshold of this virus allows it to overcome the mildly acidic endosomal pH. 

This marked ability of H5N1 to infect HTEpC-Ts prompted us to undertake functional analyses of the H5N1 HA protein which may be associated with broader tropism. Recombinant H5N3 viruses possessing the HA protein from H5N1 were able to infect HTEpC-T clone LA-9 as were wild-type H5N1 viruses (Figure 5). These results led us to surmise that the H5N1 HA protein plays an important role in broadening cell tropism to include HTEpCs as was described previously for human epithelial cells lining the bronchioles [34]. Beare et al. [64] reported that non-zoonotic AIVs (not the H5 subtype) caused mild or no clinical symptoms in experimentally infected human volunteers, and they detected a 4-fold or greater increase in HI titer in only a few volunteers. This resistance to AIV infection could be ascribed to their possession of a stable HA protein because non-zoonotic AIVs have a lower pH threshold for HA-mediated membrane fusion [34]. It may be that a different outcome would be observed if AIVs of other subtypes, such as H5N1 (which has a higher pH threshold), were used in such an experiment. However, conducting an experiment in which human volunteers are experimentally infected with harmful viruses, including H5N1, is unlikely because of ethical concerns. It would be interesting to examine possible relationships between the viral infection of HTEpC-T clones and the pH threshold for HA-mediated membrane fusion in other AIV strains (H5N6, H6N1, H7N2, H7N3, H7N4, H7N7, H7N9, H9N2, H10N7, and H10N8) that can be transmitted directly from birds to humans [1,2,3,4,5,6,7,8,9,10,11,12,13,14,15,16].

Recent reports, including our own, have shown that the acid stability of the H5N1 HA protein is more vulnerable to pH (i.e., the pH threshold of H5N1 viruses is higher) than that of non-zoonotic AIVs [34,65,66,67,68,69,70,71]. After viral internalization via endocytosis, this biochemical property (i.e., acid stability) of H5N1 may support infectivity as shown by the results in human tracheal (present results) and bronchiolar (previous results) epithelial cells [34]. An acid-destabilized HA protein of H5N1 could increase the infection of target cells once the virus enters the target cell; by contrast, acid destabilization is likely to disturb their persistence in host environments. Usually, AIVs replicate in the intestinal tract of waterfowl [72,73,74,75]. The contents of the avian intestine are relatively acidic, although there are slight differences among species [76,77,78,79]. In this case, AIVs possessing acid-stable HA molecules (e.g., the H5N3 and H5N9 strains used in this study) have an advantage in that they can persist in the intestinal tract of waterfowl, although the degree to which viruses infect intestinal cells is another aspect to consider. Further studies are required to reveal the endosomal environment of avian intestinal cells and the relationship between their endosomal pH values and susceptibility to infection by AIVs. Should AIVs find their way into the human airway after close contact with birds, then the virus may attach to the surface of airway epithelial cells. Indeed, the human nasal mucosa is relatively acidic [80,81,82]. Such an acidic host environment (e.g., the avian intestinal tract and the human upper airway) may prevent H5N1 from persisting on the surface of host cells, although the virus can replicate efficiently once it has entered the target cell. This might be one reason that cases of human infection by H5N1 have been sporadic to date, although human infection by this virus is more common in some areas including Egypt. 

We also evaluated the susceptibility of primary HTEpCs to infection by AIVs. The susceptibility of primary HTEpCs to infection by AIVs broadly reflected that of HTEpC-Ts (Figure 1, Figure 5 and Figure 6), suggesting that HTEpC-Ts can be used as a cell model for the detailed analysis of AIV infection of human conducting airways. Regarding the susceptibility of primary HTEpCs to infection by AIVs, the antigen-positive/negative ratio of AIVs in HTEpCs was similar to that in HTEpC-T clones, although this ratio for non-zoonotic AIVs in primary HTEpCs (especially in cells infected at an m.o.i. of 10) was different from that observed in HTEpC-T clones (i.e., primary HTEpCs were more susceptible than HTEpC-T clones to infection by non-zoonotic AIVs) (Figure 1A–C, Figure 5, and Figure 6A,B). These phenomena suggest two possibilities with respect to the endosomal pH in primary HTEpCs. First, we failed to generate HTEpC-T clones with an acidic endosomal pH, although primary HTEpCs may also include cells with an acidic endosomal pH. Second, the pH in primary HTEpCs is often mildly acidic; however, endogenous proteases may support viral infection of HTEpCs because tracheal proteases, such as transmembrane protease serine 2 (TMPRSS2) [83] and human airway trypsin-like protease (HAT) [84], which are required to process released virions, allow for multiple cycles of viral infection [85]. Further studies should generate more than six HTEpC-T clones and analyze both the endosomal pH and susceptibility to infection by AIVs. 

Even if the difference in infectivity between H5N1 and non-zoonotic AIVs in primary HTEpCs is not as marked as that in HTEpC-Ts, the difference seems to reflect the amount of virions released from infected HTEpCs upon long-term cultivation at a low m.o.i; this is because influenza viruses exhibit exponential growth as a result of multiple cycles of viral infection during long-term cultivation. During the long-term cultivation of virus within primary HTEpCs, the amount of H5N1 virions released at each time point was significantly higher than the amount of non-zoonotic AIV virions (Figure 7). In addition, the replication kinetics of recombinant Dk/Hk (H5N3) harboring H5N1 HA genes were similar to those of the H5N1 virus (Figure 7). These results suggest that the H5N1 virus can replicate continuously, because it has a higher pH threshold for membrane fusion; it can then spread to the entire tracheal region within the respiratory tract. This could be one mechanism underlying H5N1 pathogenesis in human airway epithelia.

## 5. Conclusions

Because viral replication in the tracheal region of the respiratory tract may result in infection of the terminal part of the LRT (i.e., bronchioles/alveoli) which can result in ARDS, it is important to know the degree to which H5N1 infects tracheal epithelial cells. The results presented herein suggest that the susceptibility of tracheal epithelial cells to infection by H5N1 is determined by the balance between the acid stability of the HA molecule (i.e., pH threshold of HA-mediated membrane fusion) and the pH of the endosomal compartment. Further research into the HA-governed mechanisms underlying membrane fusion will help us to develop effective strategies for the surveillance and control of AIVs.

## Supplementary Materials

The following are available online at https://www.mdpi.com/1999-4915/12/1/82/s1. Figure S1. Establishment of human tracheal epithelial cell clones. Figure S2. Viral antigen expression in human tracheal epithelial cell clones. Figure S3. Uncropped images of the Western blots in Figure 3. Figure S4. Graphs showing the correlation between copy number and infectious titer of virus particles released from infected HTEpCs. Table S1. Summary of the pH thresholds for viral–cell membrane fusion for all AIV strains used in this study.

## References

[1] Chen H., Yuan H., Gao R., Zhang J., Wang D., Xiong Y., Fan G., Yang F., Li X., Zhou J. (2014). Clinical and epidemiological characteristics of a fatal case of avian influenza A H10N8 virus infection: A descriptive study. Lancet.

[2] Fouchier R.A., Schneeberger P.M., Rozendaal F.W., Broekman J.M., Kemink S.A., Munster V., Kuiken T., Rimmelzwaan G.F., Schutten M., van Doornum G.J. (2004). Avian influenza A virus (H7N7) associated with human conjunctivitis and a fatal case of acute respiratory distress syndrome. Proc. Natl. Acad. Sci. USA.

[3] Kalthoff D., Globig A., Beer M. (2010). (Highly pathogenic) avian influenza as a zoonotic agent. Vet. Microbiol..

[4] Lin Y.P., Shaw M., Gregory V., Cameron K., Lim W., Klimov A., Subbarao K., Guan Y., Krauss S., Shortridge K. (2000). Avian-to-human transmission of H9N2 subtype influenza A viruses: Relationship between H9N2 and H5N1 human isolates. Proc. Natl. Acad. Sci. USA.

[5] Su S., Bi Y., Wong G., Gray G.C., Gao G.F., Li S. (2015). Epidemiology, Evolution, and Recent Outbreaks of Avian Influenza Virus in China. J. Virol..

[6] Yang Z.F., Mok C.K., Peiris J.S., Zhong N.S. (2015). Human Infection with a Novel Avian Influenza A (H5N6) Virus. N. Engl. J. Med..

[7] Yu H., Cowling B.J., Feng L., Lau E.H., Liao Q., Tsang T.K., Peng Z., Wu P., Liu F., Fang V.J. (2013). Human infection with avian influenza A H7N9 virus: An assessment of clinical severity. Lancet.

[8] Arzey G.G., Kirkland P.D., Arzey K.E., Frost M., Maywood P., Conaty S., Hurt A.C., Deng Y.M., Iannello P., Barr I. (2012). Influenza virus A (H10N7) in chickens and poultry abattoir workers, Australia. Emerg. Infect. Dis..

[9] Peiris M., Yuen K.Y., Leung C.W., Chan K.H., Ip P.L., Lai R.W., Orr W.K., Shortridge K.F. (1999). Human infection with influenza H9N2. Lancet.

[10] Tweed S.A., Skowronski D.M., David S.T., Larder A., Petric M., Lees W., Li Y., Katz J., Krajden M., Tellier R. (2004). Human illness from avian influenza H7N3, British Columbia. Emerg. Infect. Dis..

[11] Lopez-Martinez I., Balish A., Barrera-Badillo G., Jones J., Nuñez-García T.E., Jang Y., Aparicio-Antonio R., Azziz-Baumgartner E., Belser J.A., Ramirez-Gonzalez J.E. (2013). Highly pathogenic avian influenza A (H7N3) virus in poultry workers, Mexico, 2012. Emerg. Infect. Dis..

[12] Ostrowsky B., Huang A., Terry W., Anton D., Brunagel B., Traynor L., Abid S., Johnson G., Kacica M., Katz J. (2012). Low pathogenic avian influenza A (H7N2) virus infection in immunocompromised adult, New York, USA, 2003. Emerg. Infect. Dis..

[13] Yuan J., Zhang L., Kan X., Jiang L., Yang J., Guo Z., Ren Q. (2013). Origin and molecular characteristics of a novel 2013 avian influenza A (H6N1) virus causing human infection in Taiwan. Clin. Infect. Dis..

[14] Gao P., Du H., Fan L., Chen L., Liao M., Xu C., Xiang B., Ren T. (2018). Human infection with an avian-origin influenza A (H7N4) virus in Jiangsu: A potential threat to China. J. Infect..

[15] Koopmans M., Wilbrink B., Conyn M., Natrop G., van der Nat H., Vennema H., Meijer A., van Steenbergen J., Fouchier R., Osterhaus A. (2004). Transmission of H7N7 avian influenza A virus to human beings during a large outbreak in commercial poultry farms in the Netherlands. Lancet.

[16] Tong X.C., Weng S.S., Xue F., Wu X., Xu T.M., Zhang W.H. (2018). First human infection by a novel avian influenza A (H7N4) virus. J. Infect..

[17] Shinya K., Ebina M., Yamada S., Ono M., Kasai N., Kawaoka Y. (2006). Avian flu: Influenza virus receptors in the human airway. Nature.

[18] Van Riel D., Munster V.J., de Wit E., Rimmelzwaan G.F., Fouchier R.A., Osterhaus A.D., Kuiken T. (2006). H5N1 Virus Attachment to Lower Respiratory Tract. Science.

[19] De Jong M.D., Simmons C.P., Thanh T.T., Hien V.M., Smith G.J., Chau T.N.B., Hoang D.M., Chau N.V.V., Khanh T.H., Dong V.C. (2006). Fatal outcome of human influenza A (H5N1) is associated with high viral load and hypercytokinemia. Natl. Med..

[20] Gu J., Xie Z., Gao Z., Liu J., Korteweg C., Ye J., Lau L.T., Lu J., Gao Z., Zhang B. (2007). H5N1 infection of the respiratory tract and beyond: A molecular pathology study. Lancet.

[21] Kandun I.N., Wibisono H., Sedyaningsih E.R., Hadisoedarsuno W., Purba W., Santoso H., Septiawati C., Tresnaningsih E., Heriyanto B., Yuwono D. (2006). Three Indonesian clusters of H5N1 virus infection in 2005. N. Engl. J. Med..

[22] Korteweg C., Gu J. (2008). Pathology, molecular biology, and pathogenesis of avian influenza A (H5N1) infection in humans. Am. J. Pathol..

[23] Oner A.F., Bay A., Arslan S., Akdeniz H., Sahin H.A., Cesur Y., Epcacan S., Yilmaz N., Deger I., Kizilyildiz B. (2006). Avian influenza A (H5N1) infection in eastern Turkey in 2006. N. Engl. J. Med..

[24] Uiprasertkul M., Kitphati R., Puthavathana P., Kriwong R., Kongchanagul A., Ungchusak K., Angkasekwinai S., Chokephaibulkit K., Srisook K., Vanprapar N. (2007). Apoptosis and pathogenesis of avian influenza A (H5N1) virus in humans. Emerg. Infect. Dis..

[25] Shu Y., Yu H., Li D. (2006). Lethal avian influenza A (H5N1) infection in a pregnant woman in Anhui Province, China. N. Engl. J. Med..

[26] Gaush C.R., Smith T.F. (1968). Replication and plaque assay of influenza virus in an established line of canine kidney cells. Appl. Microbiol..

[27] Govorkova E.A., Kodihalli S., Alymova I.V., Fanget B., Webster R.G. (1999). Growth and immunogenicity of influenza viruses cultivated in Vero or MDCK cells and in embryonated chicken eggs. Dev. Biol. Stand..

[28] Meguro H., Bryant J.D., Torrence A.E., Wright P.F. (1979). Canine kidney cell line for isolation of respiratory viruses. J. Clin. Microbiol..

[29] Schepetiuk S.K., Kok T. (1993). The use of MDCK, MEK and LLC-MK2 cell lines with enzyme immunoassay for the isolation of influenza and parainfluenza viruses from clinical specimens. J. Virol. Methods.

[30] Yamada S., Hatta M., Staker B.L., Watanabe S., Imai M., Shinya K., Sakai-Tagawa Y., Ito M., Ozawa M., Watanabe T. (2010). Biological and structural characterization of a host-adapting amino acid in influenza virus. PLoS Pathog..

[31] Wang C., Lee H.H., Yang Z.F., Mok C.K., Zhang Z. (2016). PB2-Q591K Mutation Determines the Pathogenicity of Avian H9N2 Influenza Viruses for Mammalian Species. PLoS ONE.

[32] Watanabe Y., Ibrahim M.S., Ellakany H.F., Kawashita N., Mizuike R., Hiramatsu H., Sriwilaijaroen N., Takagi T., Suzuki Y., Ikuta K. (2011). Acquisition of human-type receptor binding specificity by new H5N1 influenza virus sublineages during their emergence in birds in Egypt. PLoS Pathog..

[33] Watanabe Y., Arai Y., Daidoji T., Kawashita N., Ibrahim M.S., El-Gendy E.E.D.M., Hiramatsu H., Kubota-Koketsu R., Takagi T., Murata T. (2015). Characterization of H5N1 influenza virus variants with hemagglutinin mutations isolated from patients. MBio.

[34] Daidoji T., Watanabe Y., Ibrahim M.S., Yasugi M., Maruyama H., Masuda T., Arai F., Ohba T., Honda A., Ikuta K. (2015). Avian Influenza Virus Infection of Immortalized Human Respiratory Epithelial Cells Depends upon a Delicate Balance between Hemagglutinin Acid Stability and Endosomal pH. J. Biol. Chem..

[35] Di Lonardo A., Buttinelli G., Amato C., Novello F., Ridolfi B., Fiore L. (2002). Rapid methods for identification of poliovirus isolates and determination of polio neutralizing antibody titers in human sera. J. Virol. Methods.

[36] Basler C.F., Reid A.H., Dybing J.K., Janczewski T.A., Fanning T.G., Zheng H., Salvatore M., Perdue M.L., Swayne D.E., Garcia-Sastre A. (2001). Sequence of the 1918 pandemic influenza virus nonstructural gene (NS) segment and characterization of recombinant viruses bearing the 1918 NS genes. Proc. Natl. Acad. Sci. USA.

[37] Fodor E., Devenish L., Engelhardt O.G., Palese P., Brownlee G.G., Garcia-Sastre A. (1999). Rescue of influenza A virus from recombinant DNA. J. Virol..

[38] Tumpey T.M., Garcia-Sastre A., Mikulasova A., Taubenberger J.K., Swayne D.E., Palese P., Basler C.F. (2002). Existing antivirals are effective against influenza viruses with genes from the 1918 pandemic virus. Proc. Natl. Acad. Sci. USA.

[39] Daidoji T., Koma T., Du A., Yang C.S., Ueda M., Ikuta K., Nakaya T. (2008). H5N1 avian influenza virus induces apoptotic cell death in mammalian airway epithelial cells. J. Virol..

[40] Arai Y., Kawashita N., Daidoji T., Ibrahim M.S., El-Gendy E.M., Takagi T., Takahashi K., Suzuki Y., Ikuta K., Nakaya T. (2016). Novel Polymerase Gene Mutations for Human Adaptation in Clinical Isolates of Avian H5N1 Influenza Viruses. PLoS Pathog..

[41] Lee C.W., Jung K., Jadhao S.J., Suarez D.L. (2008). Evaluation of chicken-origin (DF-1) and quail-origin (QT-6) fibroblast cell lines for replication of avian influenza viruses. J. Virol. Methods.

[42] Garten W., Klenk H.D. (1999). Understanding influenza virus pathogenicity. Trends Microbiol..

[43] Hatta M., Gao P., Halfmann P., Kawaoka Y. (2001). Molecular basis for high virulence of Hong Kong H5N1 influenza A viruses. Science.

[44] Nobusawa E., Aoyama T., Kato H., Suzuki Y., Tateno Y., Nakajima K. (1991). Comparison of complete amino acid sequences and receptor-binding properties among 13 serotypes of hemagglutinins of influenza A viruses. Virology.

[45] Lakadamyali M., Rust M.J., Zhuang X. (2004). Endocytosis of influenza viruses. Microbes Infect..

[46] Pelkmans L., Helenius A. (2003). Insider information: What viruses tell us about endocytosis. Curr. Opin. Cell Biol..

[47] Sieczkarski S.B., Whittaker G.R. (2003). Differential requirements of Rab5 and Rab7 for endocytosis of influenza and other enveloped viruses. Traffic.

[48] Akita H., Ito R., Khalil I.A., Futaki S., Harashima H. (2004). Quantitative three-dimensional analysis of the intracellular trafficking of plasmid DNA transfected by a nonviral gene delivery system using confocal laser scanning microscopy. Mol. Ther. J. Am. Soc. Gene Ther..

[49] Teichgräber V., Ulrich M., Endlich N., Riethmüller J., Wilker B., De Oliveira–Munding C.C., Van Heeckeren A.M., Barr M.L., Von Kürthy G., Schmid K.W. (2008). Ceramide accumulation mediates inflammation, cell death and infection susceptibility in cystic fibrosis. Natl. Med..

[50] Zhang Y., Li X., Grassme H., Doring G., Gulbins E. (2010). Alterations in ceramide concentration and pH determine the release of reactive oxygen species by Cftr-deficient macrophages on infection. J. Immunol..

[51] Matrosovich M.N., Matrosovich T.Y., Gray T., Roberts N.A., Klenk H.D. (2004). Human and avian influenza viruses target different cell types in cultures of human airway epithelium. Proc. Natl. Acad. Sci. USA.

[52] Nicholls J.M., Chan M.C.W., Chan W.Y., Wong H.K., Cheung C.Y., Kwong D.L.W., Wong M.P., Chui W.H., Poon L.L.M., Tsao S.W. (2007). Tropism of avian influenza A (H5N1) in the upper and lower respiratory tract. Natl. Med..

[53] Van Riel D., Leijten L.M., de Graaf M., Siegers J.Y., Short K.R., Spronken M.I., Schrauwen E.J., Fouchier R.A., Osterhaus A.D., Kuiken T. (2013). Novel avian-origin influenza A (H7N9) virus attaches to epithelium in both upper and lower respiratory tract of humans. Am. J. Pathol..

[54] Van Riel D., Munster V.J., de Wit E., Rimmelzwaan G.F., Fouchier R.A., Osterhaus A.D., Kuiken T. (2007). Human and avian influenza viruses target different cells in the lower respiratory tract of humans and other mammals. Am. J. Pathol..

[55] Yao L., Korteweg C., Hsueh W., Gu J. (2008). Avian influenza receptor expression in H5N1-infected and noninfected human tissues. FASEB J..

[56] Chan R.W., Chan M.C., Nicholls J.M., Malik Peiris J.S. (2013). Use of ex vivo and in vitro cultures of the human respiratory tract to study the tropism and host responses of highly pathogenic avian influenza A (H5N1) and other influenza viruses. Virus Res..

[57] Nicholls J.M., Bourne A.J., Chen H., Guan Y., Peiris J.S. (2007). Sialic acid receptor detection in the human respiratory tract: Evidence for widespread distribution of potential binding sites for human and avian influenza viruses. Respir. Res..

[58] Nicholls J.M., Chan R.W., Russell R.J., Air G.M., Peiris J.S. (2008). Evolving complexities of influenza virus and its receptors. Trends Microbiol..

[59] Walther T., Karamanska R., Chan R.W., Chan M.C., Jia N., Air G., Hopton C., Wong M.P., Dell A., Peiris J.M. (2013). Glycomic analysis of human respiratory tract tissues and correlation with influenza virus infection. PLoS Pathog..

[60] Fulcher M.L., Gabriel S., Burns K.A., Yankaskas J.R., Randell S.H. (2005). Well-differentiated human airway epithelial cell cultures. Methods Mol. Med..

[61] Prytherch Z., Job C., Marshall H., Oreffo V., Foster M., BeruBe K. (2011). Tissue-Specific stem cell differentiation in an in vitro airway model. Macromol. Biosci..

[62] Rowe R.K., Brody S.L., Pekosz A. (2004). Differentiated cultures of primary hamster tracheal airway epithelial cells. Vitro Cell. Dev. Biol. Anim..

[63] You Y., Richer E.J., Huang T., Brody S.L. (2002). Growth and differentiation of mouse tracheal epithelial cells: Selection of a proliferative population. Am. J. Physiol. Lung Cell. Mol. Physiol..

[64] Beare A.S., Webster R.G. (1991). Replication of avian influenza viruses in humans. Arch. Virol..

[65] Byrd-Leotis L., Galloway S.E., Agbogu E., Steinhauer D.A. (2015). Influenza hemagglutinin (HA) stem region mutations that stabilize or destabilize the structure of multiple HA subtypes. J. Virol..

[66] DuBois R.M., Zaraket H., Reddivari M., Heath R.J., White S.W., Russell C.J. (2011). Acid stability of the hemagglutinin protein regulates H5N1 influenza virus pathogenicity. PLoS Pathog..

[67] Galloway S.E., Reed M.L., Russell C.J., Steinhauer D.A. (2013). Influenza HA subtypes demonstrate divergent phenotypes for cleavage activation and pH of fusion: Implications for host range and adaptation. PLoS Pathog..

[68] Okamatsu M., Motohashi Y., Hiono T., Tamura T., Nagaya K., Matsuno K., Sakoda Y., Kida H. (2016). Is the optimal pH for membrane fusion in host cells by avian influenza viruses related to host range and pathogenicity?. Arch. Virol..

[69] Reed M.L., Bridges O.A., Seiler P., Kim J.K., Yen H.L., Salomon R., Govorkova E.A., Webster R.G., Russell C.J. (2010). The pH of activation of the hemagglutinin protein regulates H5N1 influenza virus pathogenicity and transmissibility in ducks. J. Virol..

[70] Reed M.L., Yen H.L., DuBois R.M., Bridges O.A., Salomon R., Webster R.G., Russell C.J. (2009). Amino acid residues in the fusion peptide pocket regulate the pH of activation of the H5N1 influenza virus hemagglutinin protein. J. Virol..

[71] Zaraket H., Bridges O.A., Duan S., Baranovich T., Yoon S.W., Reed M.L., Salomon R., Webby R.J., Webster R.G., Russell C.J. (2013). Increased acid stability of the hemagglutinin protein enhances H5N1 influenza virus growth in the upper respiratory tract but is insufficient for transmission in ferrets. J. Virol..

[72] Horimoto T., Kawaoka Y. (2005). Influenza: Lessons from past pandemics, warnings from current incidents. Natl. Rev. Microbiol..

[73] Kida H., Yanagawa R., Matsuoka Y. (1980). Duck influenza lacking evidence of disease signs and immune response. Infect. Immun..

[74] Kim J.K., Negovetich N.J., Forrest H.L., Webster R.G. (2009). Ducks: The "Trojan horses" of H5N1 influenza. Influenza Other Respir. Viruses.

[75] Webster R.G., Yakhno M., Hinshaw V.S., Bean W.J., Murti K.G. (1978). Intestinal influenza: Replication and characterization of influenza viruses in ducks. Virology.

[76] Bowen T.E., Waldroup P.W. (1969). The influence of propylene glycol on pH of the gastrointestinal tract and the incidence of leg abnormalities in broiler chicks. Poult. Sci..

[77] Farner D.S., and Seaman E. (1942). The hydrogen ion concentration in avian digestive tracts. Poult. Sci..

[78] Hewitt E.A., Schelkopf R.L. (1955). PH values and enzymatic activity of the digestive tract of the chicken. Am. J. Vet. Res..

[79] Lin G.L., Himes J.A., Cornelius C.E. (1974). Bilirubin and biliverdin excretion by the chicken. Am. J. Physiol..

[80] Fischer H., Widdicombe J.H. (2006). Mechanisms of acid and base secretion by the airway epithelium. J. Membr. Biol..

[81] Russell C.J., Hu M., Okda F.A. (2018). Influenza Hemagglutinin Protein Stability, Activation, and Pandemic Risk. Trends Microbiol..

[82] Washington N., Steele R.J., Jackson S.J., Bush D., Mason J., Gill D.A., Pitt K., Rawlins D.A. (2000). Determination of baseline human nasal pH and the effect of intranasally administered buffers. Int. J. Pharm..

[83] Wilson S., Greer B., Hooper J., Zijlstra A., Walker B., Quigley J., Hawthorne S. (2005). The membrane-anchored serine protease, TMPRSS2, activates PAR-2 in prostate cancer cells. Biochem. J..

[84] Yasuoka S., Ohnishi T., Kawano S., Tsuchihashi S., Ogawara M., Masuda K., Yamaoka K., Takahashi M., Sano T. (1997). Purification, characterization, and localization of a novel trypsin-like protease found in the human airway. Am. J. Respir. Cell Mol. Biol..

[85] Böttcher E., Matrosovich T., Beyerle M., Klenk H.D., Garten W., Matrosovich M. (2006). Proteolytic activation of influenza viruses by serine proteases TMPRSS2 and HAT from human airway epithelium. J. Virol..

