# Supplementary material for: Infection of Human Tracheal Epithelial Cells by H5 Avian Influenza Virus Is Regulated by the Acid Stability of Hemagglutinin and the pH of Target Cell Endosomes
Source: Viruses. 2020 Jan 9;12(1):82. doi: 10.3390/v12010082 (PMC7019350; doi:10.3390/v12010082)
Supplement: Supplementary file 1 [file viruses-12-00082-s001.zip › viruses-690496_Supplemental Figures.v1.pdf]

Supplementary Information

Infection of human tracheal epithelial cells by H5 avian influenza virus is regulated by the acid stability of hemagglutinin and the pH of target cell endosomes

Tomo Daidoji, Junichi Kajikawa, Yasuha Arai, Yohei Watanabe, Ryohei Hirose, Takaaki Nakaya

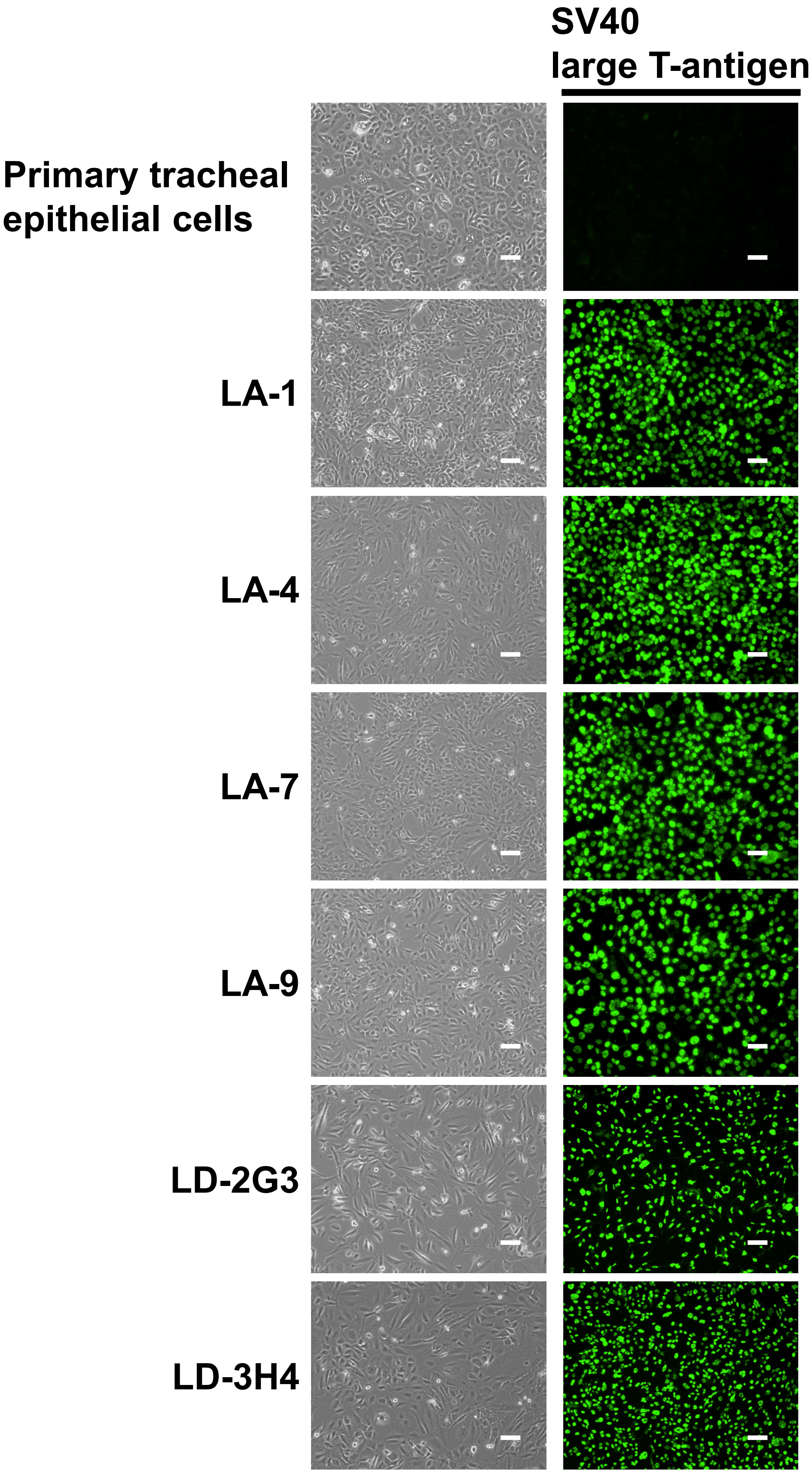

Figure S1. Establishment of human tracheal epithelial cell clones.
Morphology (left panels) and immunostaining (right panels) of primary human tracheal epithelial cells (HTEpCs; a mixed population) and their resultant clones (HTEpCs-T; LA-1, LA-4, LA-7, LA-9, LD-2G3, and LD-3H4) for expression of the SV40 large T-antigen. Scale bars, 100 μm.

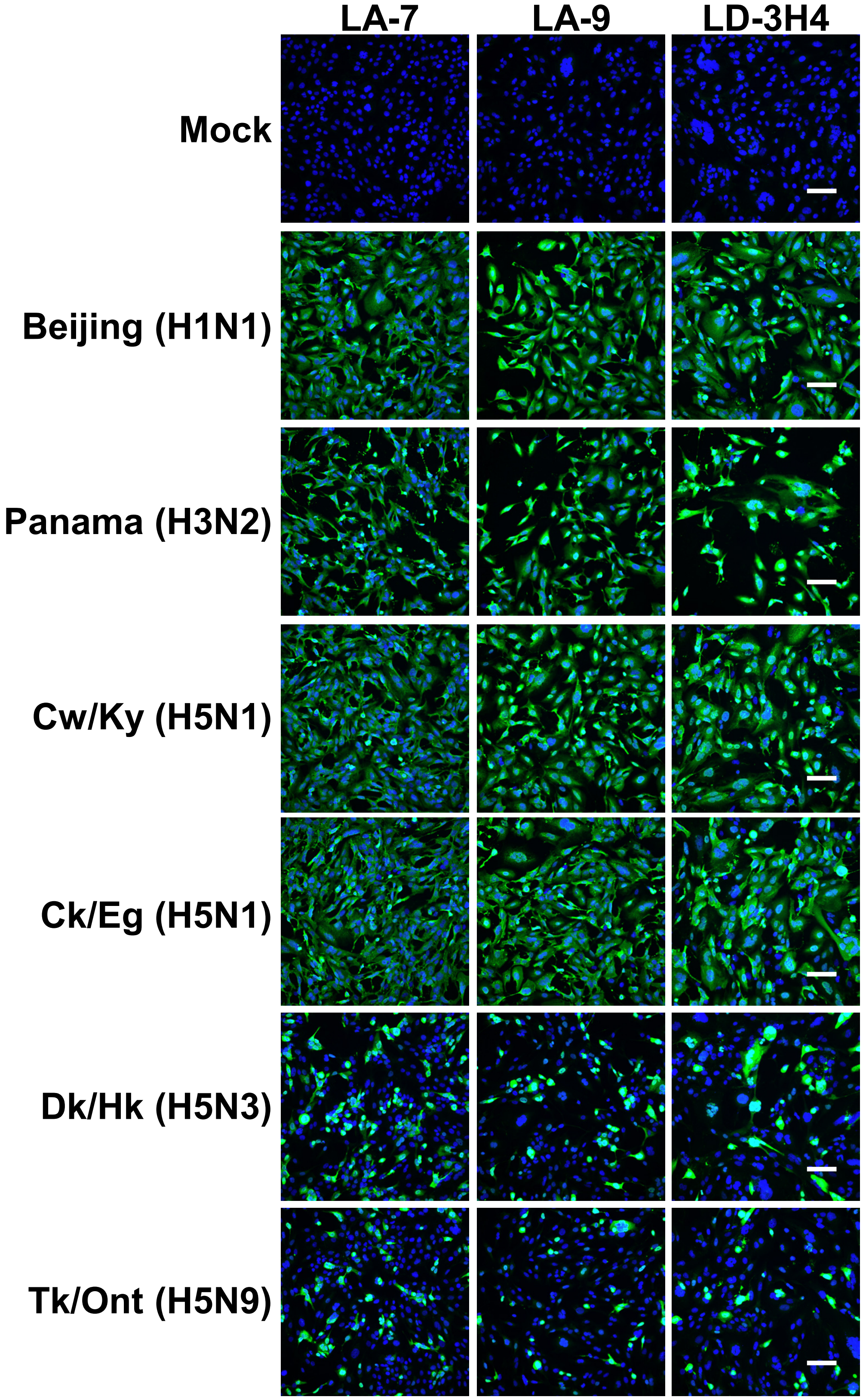

Figure S2. Viral antigen expression in human tracheal epithelial cell clones.
Representative HTEpC-T clones (LA-7, LA-9 and LD-3H4) were infected with different strains of human influenza virus: Beijing (H1N1) and Panama (H3N2); H5N1 [Cw/Ky (H5N1) and Ck/Eg (H5N1)]; and previously circulating AIVs [Dk/Hk (H5N3) and Tk/Ont (H5N9)]. All cells were infected at a multiplicity of infection of 10. Viral antigens were detected by immunostaining (see "Materials and methods") at 16 h post-infection (green). Cell nuclei were counterstained with Hoechst 33342 (blue). Scale bars, 100 μm.

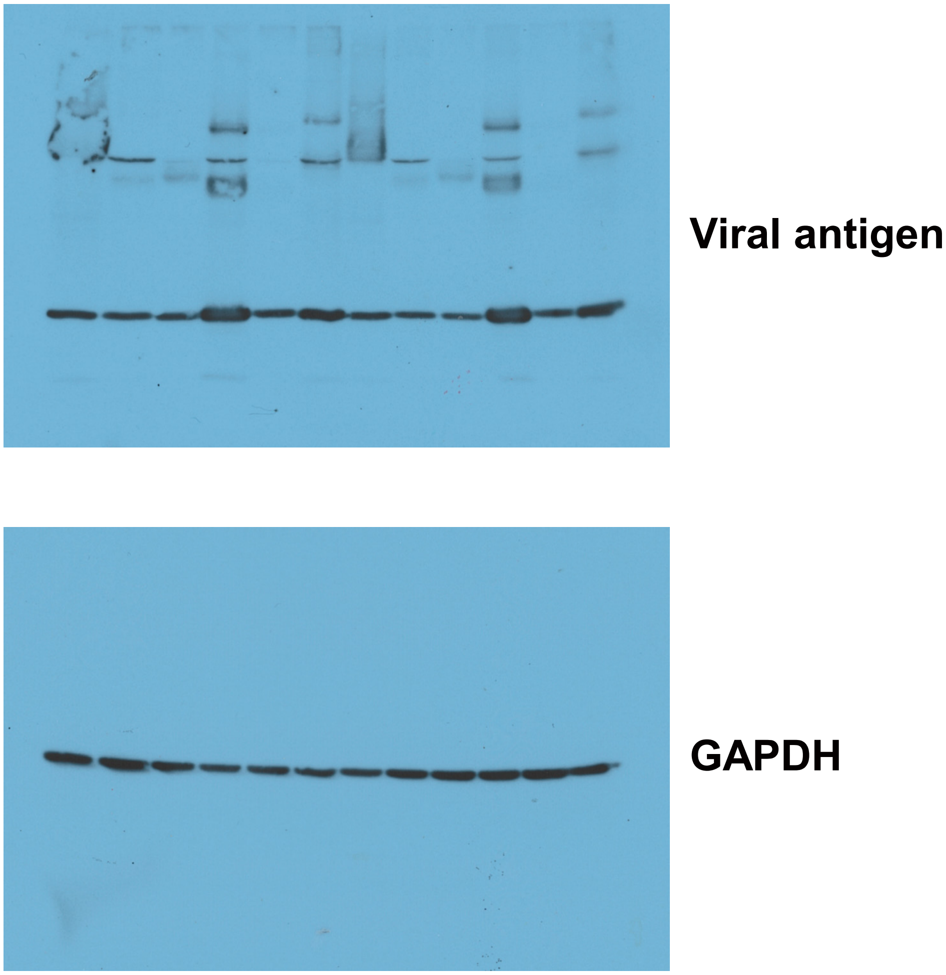

Figure S3. Uncropped images of the Western blots in Figure 3.

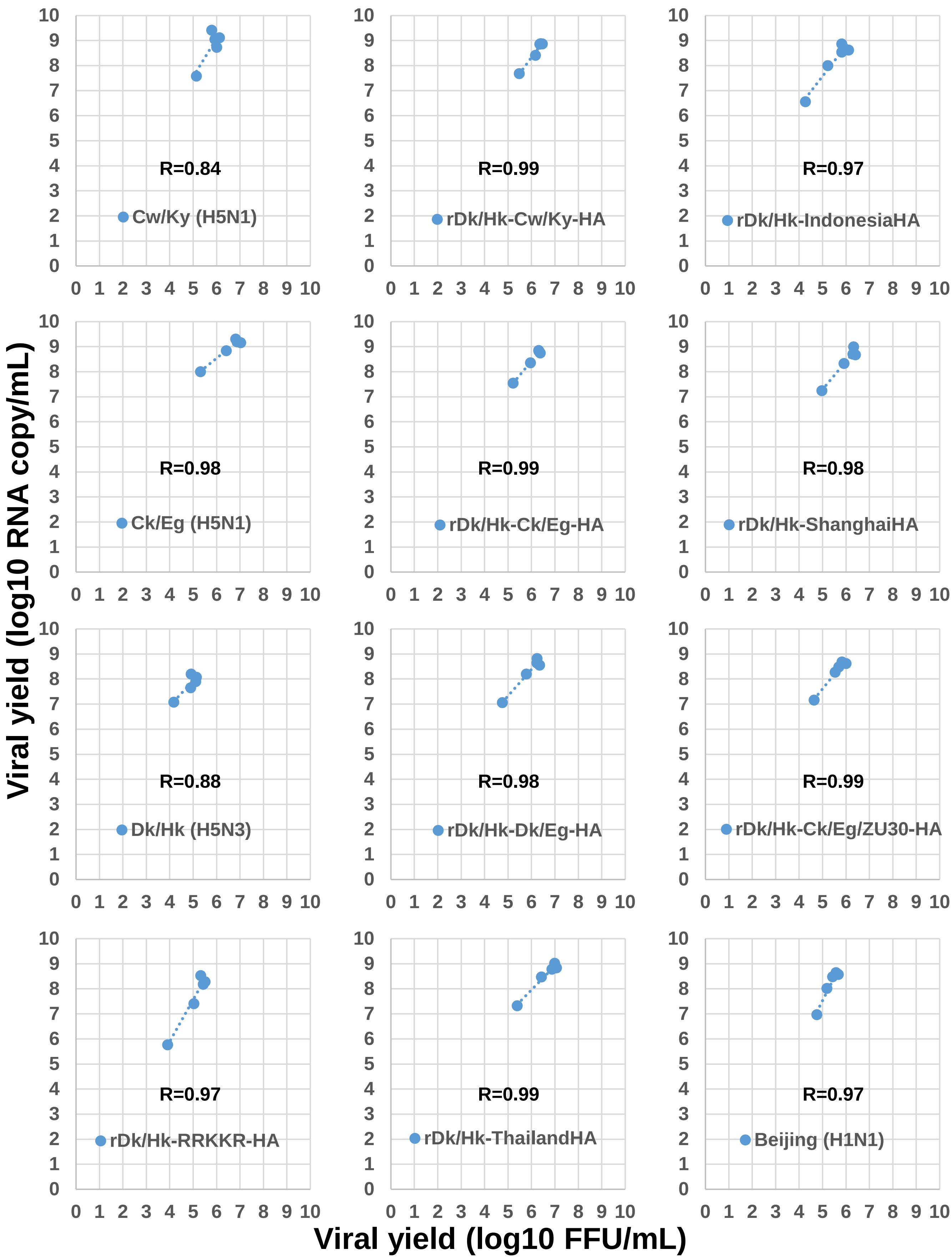

Figure S4. Graphs showing the correlation between copy number and infectious titer of virus particles released from infected HTEpCs.
Each parameter was taken from Figure 7A and 7B. The virus names are the same as those described in Figure 7.
