# Supplementary material for: Infection of Human Tracheal Epithelial Cells by H5 Avian Influenza Virus Is Regulated by the Acid Stability of Hemagglutinin and the pH of Target Cell Endosomes
Source: Viruses. 2020 Jan 9;12(1):82. doi: 10.3390/v12010082 (PMC7019350; doi:10.3390/v12010082)
Supplement: Supplementary file 1 [file viruses-12-00082-s001.zip › viruses-690496_TABLE S1.v1.pdf]

TABLE S1. Summary of the pH thresholds for viral–cell membrane fusion for all AIV strains used in this study

Viral strain	pH thresholds
A/crow/Kyoto/53/04 (H5N1)	5.625
A/chicken/Egypt/CL6/07 (H5N1)	5.625
A/duck/Egypt/D1Br12/2007 (H5N1)	n.d.
A/Thailand/Kan353/04 (H5N1)	5.75
A/Indonesia/5/05 (H5N1)	5.625
A/Shanghai/1/06 (H5N1)	5.625
A/chicken/Egypt/ZU30/2016 (H5N1)	n.d.
A/Duck/Hong Kong/820/80 (H5N3)	5.375
A/Turkey/Ontario/7732/66 (H5N9)	5.125

These parameters were originally published in Daidoji et al. (34) ©American Society for Biochemistry and Molecular Biology.
